# Critical assessment of copper-alginate hydrogel beads as recyclable and heterogeneous catalysts for aqueous azide‒alkyne cycloaddition

**DOI:** 10.3389/fchem.2025.1644592

**Published:** 2025-08-13

**Authors:** Yanina Moglie, Eduardo Buxaderas, Agoney González Cabrera, David Díaz Díaz

**Affiliations:** 1 Instituto de Química del Sur, INQUISUR (CONICET-UNS), Departamento de Química, Universidad Nacional del Sur, Bahía Blanca, Argentina; 2 AFM-NANO, Instituto Universitario de Bio-Orgánica Antonio González (IUBO-AG), Universidad de La Laguna, San Cristóbal de La Laguna, Spain; 3 Departamento de Química Orgánica, Universidad de La Laguna, San Cristóbal de La Laguna, Spain

**Keywords:** sustainable catalysis, copper alginate, click chemistry, 1,2,3-triazoles, green chemistry

## Abstract

**Introduction:**

We report a reproducible and sustainable catalytic system based on copper ion-crosslinked alginate hydrogels for the synthesis of 1,2,3-triazoles via aqueous 1,3-dipolar cycloaddition at room temperature. The catalyst, derived from a biodegradable matrix, is prepared through a simple, energy-efficient method and operates under mild, eco-friendly conditions.

**Methods:**

Copper(II)-alginate hydrogels were prepared by ionic crosslinking. Sodium ascorbate was identified as a key reducing agent to generate catalytically active Cu(I) species. Different catalyst morphologies (hydrogel, aerogel, xerogel) were evaluated. Structural and morphological characterization was performed using FT-IR, SEM, EDX, TGA, and AAS.

**Results:**

The Cu(II)-alginate hydrogel (Cu(II)-AHG) exhibited the highest performance, delivering >95% yields within 3 h using only 2 mol% catalyst. The system tolerated a broad range of terminal alkynes and operated under predominantly heterogeneous conditions. The catalyst was recyclable up to four times with minimal loss in activity.

**Discussion:**

The catalyst's efficiency is attributed to its mesoporous structure and chemical integrity. These findings support the potential of alginate-based materials as sustainable supports for transition metal catalysis and contribute to the advancement of green and circular synthetic methodologies.

## 1 Introduction

Growing environmental concerns and increasingly strict regulatory frameworks have prompted a shift in chemical research toward more sustainable methodologies (Hofer and Bigorra, 2007). Thus, green chemistry principles advocate for the use of safer solvents, energy-efficient processes, and recyclable catalysts derived from renewable and biodegradable resources (Clark, 2001). In particular, biopolymers have emerged as attractive and versatile platforms have attracted increasing attention in both the academic and industrial worlds owing to their unique properties, such as biodegradability, biocompatibility and antibacterial activity (Elnashar, 2010).

**FIGURE 1:**
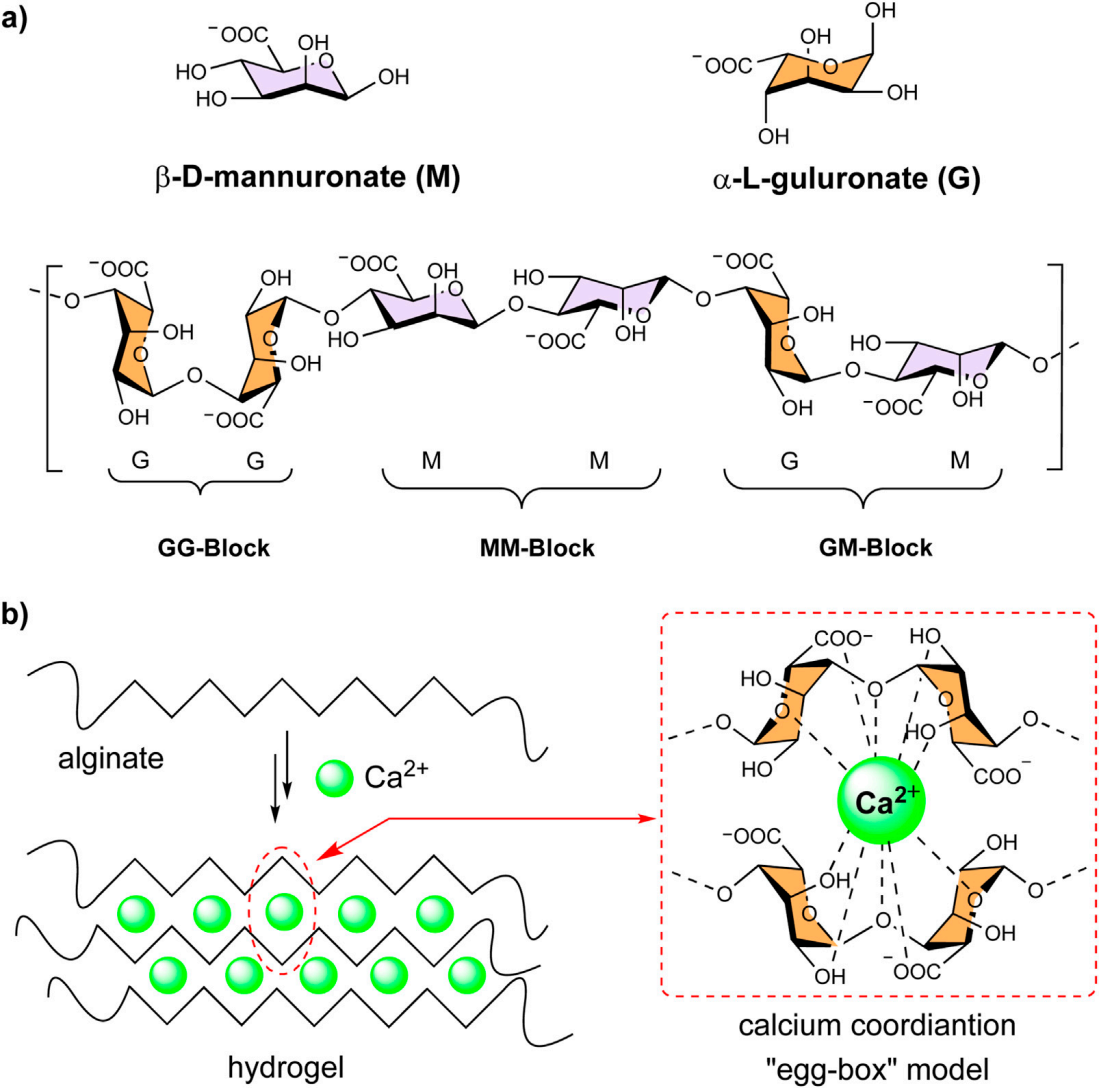
**(a)** Molecular structure of alginate. **(b)** Schematic of the egg-box structure in alginate hydrogels crosslinked with calcium ions.

In this context, biopolymers have recently gained attention in heterogeneous catalytic systems in organic synthesis as sustainable alternatives to synthetic supports (Pettignano et al., 2019). Among them, alginates -biocompatible anionic polysaccharides derived from brown seaweed-are widely used in drug delivery (Tønnesen and Karlsen, 2002), encapsulation of bioactive molecules (Kühbeck et al., 2014a; Kühbeck et al., 2014b) and as supports in biocatalysis (Sharma and Gupta, 2001; Roy and Gupta, 2004; Kumari et al., 2008; S. Zhang et al., 2014). These biopolymers are linear, unbranched copolymers made of β-D-mannuronate (M) and α-L-guluronate (G) monomers and are arranged in blocks (M)_m_, (G)_n_, and (M,G)_x_ along the chain (Figure 1). One of the key features of alginates is the ability to form robust hydrogels, with up to >95% water content, through metal ion exchange, especially with Ca^2+^ (a borderline Lewis acid) (Mendes et al., 2013). This property plays a vital role in their function as natural water storage agents in living organisms and their use in immobilization and food thickening (S. Zhang et al., 2014). The gel structure is described by the “egg-box” model, where calcium ions coordinate to the carboxylates and hydroxyls of 4 G monomers from adjacent chains (Figure 1) (Skjåk-Bræk et al., 1989; Husemann et al., 1968). The properties of alginate gels depend on the ratio and sequence of the uronic monomers (Ouverx et al., 1998), the metal ion concentration, and the maturation time (Quignard et al., 2008). Importantly, their mechanical strength can be tuned by adjusting the monomer ratio, making them versatile for different forms, such as beads, tubes, and membranes.

Owing to their unique structural versatility and eco-friendly profile, alginates have garnered significant attention in heterogeneous catalysis. Early milestones in this field include the immobilization of Pd (TPPTS) complexes on alginate aerogels for allylic substitutions by Quignard’s group (Valentin et al., 2005) and the development of Cu^2+^-alginate beads by Reddy et al. for aqueous-phase click chemistry and oxidative couplings (Reddy et al., 2007). Subsequent studies further diversified their applications, such as Guibal’s Pd-alginate/ionic liquid systems for hydrogenation (Jouannin et al., 2011), Fe^3+^-alginate gels for azo dye degradation (Dong et al., 2011), and Quignard’s bimetallic Pd-alginate aerogels for Suzuki‒Miyaura cross-couplings (Chtchigrovsky et al., 2012). Furthermore, Shi and coworkers reported the development of binary Cu–Pd–alginate dry beads for phenol hydroxylation, achieving notable catalytic activity and recyclability due to the synergistic interaction between the metal species and the alginate matrix (Shi et al., 2012). Similarly, Ag nanoparticles immobilized on alginate hydrogels have shown exceptional performance in the reduction of 4-nitrophenol, leveraging the porous structure and metal-binding capacity of the hydrogel to increase its stability and catalytic efficiency (Otari et al., 2013). Building on these foundations, recent breakthroughs have expanded the scope of alginate applications in asymmetric catalysis. For example, Pecchini et al. (2024) engineered barium alginate gel beads as heterogeneous catalysts for the enantioselective addition of indoles to nitroalkenes, achieving high yields and enantiomeric excesses under mild conditions. This work highlights the adaptability of alginate for designing chiral catalytic environments (Pecchini et al., 2024).

Despite these advancements, critical challenges persist in the field. For example, inconsistent yields of Cu-alginate systems for copper(I)-catalyzed azide‒alkyne cycloadditions (CuAAC) are often reported because of insufficient control over gelation parameters (e.g., the G/M ratio and crosslinking density) and inadequate characterization of active Cu species (Reddy et al., 2007; Bahsis et al., 2020). Furthermore, the role of alginate’s carboxylate groups in stabilizing metal nanoparticles or ions during catalysis remains poorly understood. Motivated by the need to improve robustness, reproducibility, and scalability in this area, we have revisited Cu^2+^-alginate catalytic platforms for CuAAC reactions. Our study focuses on the systematic preparation, characterization, and evaluation of copper-crosslinked alginate beads under mild aqueous conditions. By fine-tuning key variables, including reducing agent choice, catalyst morphology, and structural features of the alginate network, we report a reproducible and efficient catalytic system that operates under predominantly heterogeneous conditions and can be reused with minimal performance loss. This work contributes to the rational design of bio-based catalytic materials and provides practical insights for advancing sustainable synthetic methodologies.

## 2 Experimental section

### 2.1 Materials and methods

NMR spectra were recorded on a Bruker Avance 500 (^1^H: 400 MHz, ^13^C: 125 MHz) spectrometer. Chemical shifts for ^1^H NMR were reported as δ, parts per million (ppm), relative to the CHCl_3_ signal at 7.26 ppm. Chemical shifts for ^13^C NMR are reported as δ, ppm, relative to the signal of CDCl_3_ at 77 ppm. The coupling constants J are given in hertz (Hz). The following notations indicate the multiplicity of the signals: s = singlet, brs = broad singlet, d = doublet, t = triplet, q = quartet, dd = doublet of doublets, td = triplet of doublets, m = multiplet.

The reaction was monitored by a gas chromatograph equipped with a flame ionization detector (GC-FID, Agilent 8860) equipped with a 5%-phenyl)-methylpolysiloxane nonpolar column (HP-5 column; 30 m in length, 0.32 mm in inner diameter, 0.25 µm in film thickness). The oven temperature program was set as follows: the initial temperature (50°C) was maintained for 0.5 min. Then, the temperature was increased at a rate of 25°C/min until the final temperature (280°C) was reached, and the temperature was maintained for 13 min. The column pressure was set at 7.54 psi. The injector and detector temperatures were set to 280°C and 275°C, respectively.

TLC was facilitated by the use of the following stains in addition to UV light (254 nm) with fluorescence-indicating plates (aluminum sheets precoated with silica gel 60 F254, Merck): phosphomolybdic acid and iodine.

Elemental analysis was carried out by using a CNHS Flash EA 1112 instrument.

The copper content in the hydrogel beads was measured using an atomic absorption spectrophotometer, Varian 220 FS.

Thermogravimetric analysis (TGA) was performed by heating 10 mg of the sample from 25°C to 900°C at a heating rate of 10°C/min under a nitrogen atmosphere in a Pyris Diamond apparatus (Perkin Elmer).

The morphology of the xerogels and aerogels was observed using a ZEISS EVO 15 emission scanning electron microscope (SEM, resolution 2 nm) equipped with an energy dispersive X-ray microanalyzer (EDX) Oxford X-MAX with a 50 mm^2^ detector.

IR spectra were recorded with a Cary 360 FTIR–ATR spectrometer directly inserting a hydrogel and dried beads in the ATR probe; the spectra were recorded between 4000 and 600 cm^−1^, with a resolution of 1 cm^−1^.

N_2_ adsorption isotherms were measured on a Micromeritics ASAP 2020 apparatus on samples outgassed at 120°C. The surface areas were measured via the BET method by assuming a molecular area of N_2_ of 0.162 nm^2^. Mesopore size distributions were evaluated from the desorption curves by the correlation of Broekhoff and de Boer for cylindrical pores.

The total pore volume of the aerogels was evaluated by their mass/volume ratios. The macropore volume was calculated as the difference between the total pore volume and mesopore volume.

The analytical-grade solvents and commercially available reagents listed below were purchased from commercial suppliers and were used as received unless otherwise noted. Phenylacetylene (CAS 536-74-3, Sigma‒Aldrich), p-tolylacetylene (CAS 766-97-2), m-tolylacetylene (CAS 766-82-5), p-ethynylanisole (CAS 768-60-5), 1-ethynyl-4-fluorobenzene (CAS 766-98-3), 1-bromo-4-ethynylbenzene (CAS 766-96-1), 4-chlorophenylacetylene (CAS 873-73-4), p-ethynylbenzonitrile (CAS 3032-6), p-ethynylbenzaldehyde (CAS 63697-96-1), (4-ethynylphenyl)methanol (CAS 10602-04-7), m-ethynylpyridine (CAS 2510-23-8), prop-2-yn-1-ylbenzene (CAS 10147-11-2), pent-1-yn-3-ol (CAS 4187-86-4), 1-decyne (CAS 764-93-2), 1,3-diethynylbenzene (CAS 1785-61-1), sodium alginate (MW ≈ 380,000 g/mol, M/G ratio (25:75), CAS 9005-38-3), sodium ascorbate (CAS 134-03-2), copper chloride dihydrate (CuCl_2_·2H_2_O, CAS 10125-13-0), benzyl bromide (CAS 100-39-0), sodium azide (CAS 26628-22-8). Benzyl azide was prepared according to the literature (van Hensbergen et al., 2014). Additionally, benzyl azide solution (∼0.5 M in dichloromethane; CAS 622-79-7) and phenyl azide (∼0.5 M in *tert*-butyl methyl ether; CAS 622-37-7) were also purchased from Sigma-Aldrich.

### 2.2 Typical procedure for the preparation of copper-crosslinked alginate hydrogel beads [Cu(II)-AHG]

50 mL of a solution of sodium alginate in Milli-Q water (2% w/v) was added dropwise at room temperature to 100 mL of aqueous copper chloride solution (0.24 M, approximately neutral pH) via a dropping funnel (diameter of the tip = 4.0 mm) (Kühbeck et al., 2015). The volumetric ratio between the sodium alginate solution and the copper chloride solution (1:2) was kept constant in each case. After complete addition of the alginate solution, the beads were cured in the copper stock solution overnight at room temperature without stirring. Finally, the obtained alginate hydrogel (AHG) beads were filtered through a Büchner funnel and washed with Milli-Q water (3 × 200 mL) until no metal residues were detected in the washing solution.

### 2.3 Preparation of Cu^2+^-crosslinked alginate xerogel beads [Cu(II)-AXG]

Cu^2+^-crosslinked alginate xerogel beads were obtained by freeze-drying the corresponding hydrogel beads using a lyophilizer.

### 2.4 General procedure for the preparation of Cu^2+^-crosslinked alginate aerogel beads [Cu(II)-AAG]

Cu^2+^-crosslinked alginate hydrogel beads were first dehydrated in a series of successive ethanol–water baths with increasing alcohol concentrations (i.e., 10, 30, 50, 70, 90, and 100%) for 15 min each. The obtained alcogel beads were then dried under supercritical CO_2_ conditions (slightly above 73 bar and 31°C) using a Polaron 3100 apparatus, affording the corresponding aerogel beads.

### 2.5 General procedure for the synthesis of benzylazide

NaN_3_ (1.0 g, 15 mmol) was added to a round-bottom flask equipped with a magnetic stir bar. A 4:1 mixture of acetone and water (25 mL) was added to this mixture. Benzyl bromide (1.2 mL, 10 mmol) was added dropwise, and the mixture was stirred at room temperature for 24 h. The crude product was extracted with CH_2_Cl_2_ (2 × 25 mL), and the combined organic layers were dried over MgSO_4_. After filtration, the solvent was removed under reduced pressure, yielding benzyl azide as a light-yellow oil (1.2 g, 90% yield).

### 2.6 General procedure for the synthesis of 1,4-disubstituted 1,2,3-triazoles

The reaction tube was charged with Cu(II)-AHG (2 mol%, 5 beads), water (2 mL), and a solution of sodium ascorbate (7 mol%). Importantly, the ascorbate solution must be freshly prepared prior to use. Benzyl or phenyl azide (0.3 mmol) and the corresponding terminal alkyne (0.3 mmol) were then added to the mixture. The mixture was stirred at room temperature and monitored by TLC and/or GC-FID until complete conversion of the starting materials was achieved. Water (10 mL) was added to the reaction mixture, followed by extraction with DCM (3 × 10 mL). The combined organic phases were dried over anhydrous MgSO_4_, and the solvent was removed under vacuum to afford the crude product, which was purified by recrystallization if necessary.

### 2.7 Recovery and reuse of the catalyst

The Cu(II)-AHG beads were separated by simple filtration and washed with DCM (3 × 10 mL) and water (3 × 10 mL). The catalyst was recovered and reused in the next reaction cycle without any prior treatment. Nevertheless, fresh sodium ascorbate was required at the start of each catalytic run.

## 3 Results and discussion

The preparation of copper-ion crosslinked alginate hydrogel (Cu(II)-AHG) beads is based on the gelation method. The formation of sodium alginate (SA) gels is initiated by the introduction of copper ions. Specifically, SA powder is mixed with deionized water to form a viscous solution, which is then added dropwise into a copper (II) solution to induce the formation of Cu(II)-AHG beads (Dodero et al., 2019).

The beads were allowed to mature overnight at room temperature within the gel mixture. The samples were subsequently filtered and thoroughly rinsed with distilled water to remove excess metal cations and counterions. This ionotropic gelation process is both spontaneous and environmentally friendly, as it does not involve the use of toxic agents.

Dry forms of alginate gels are obtained through drying methods such as oven drying, freeze-drying, or supercritical drying (Di Renzo et al., 2006). The freeze‒drying method is employed to produce porous xerogels with three-dimensional (3D) honeycomb-like structures, which are formed by removing ice templates. This preparation process consists of two main steps: (a) freezing the hydrogel in its wet state to achieve a solid form and (b) removing the ice templates through sublimation. Compared with hydrogels, the resulting aerogel features open pores, a large specific surface area, and more active sites because of the development of macroporous and microporous 3D structures.

Aerogels are produced through drying under supercritical conditions, a process that effectively preserves the open-pore structure of the initial hydrogel. The absence of a liquid‒gas meniscus—and consequently, surface tension—during supercritical drying prevents the collapse of the delicate network structure. This yields materials with a well-defined architecture, featuring mesopores, small macropores, and exceptionally high specific surface areas, often reaching several hundred square meters per gram (m^2^/g) (Partap et al., 2006).

### 3.1 Characterization of Cu(II)-alginate beads

**FIGURE 2:**
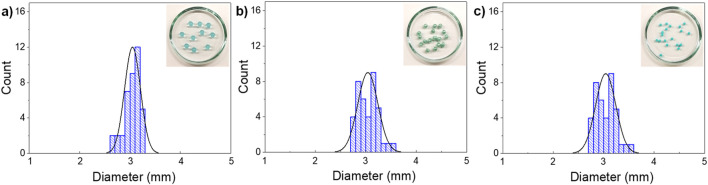
Size distribution histograms determined for **(a)** Cu(II)-AHG beads, **(b)** Cu(II)-AXG beads and **(c)** Cu(II)-AAG beads.

Copper-ion crosslinked alginate hydrogel beads [Cu(II-AHG)] were prepared following the procedure described above, and the beads exhibited a spherical shape and a characteristic blue color (see Supplementary Figure S3). Cu(II)-AHG beads were dried via freeze‒drying, resulting in the corresponding bluish-greenish xerogel beads (Cu(II)-AXG). In addition, pale blue copper (II)-aerogel beads (Cu(II)-AAG) were obtained from the corresponding alcogel beads upon drying under supercritical CO_2_ conditions. The average diameters of the different biocatalysts were 3.05 ± 0.16 mm, 3.04 ± 0.20 mm, and 1.84 ± 0.11 mm for the Cu(II)-AHG, Cu(II)-AXG, and Cu(II)-AAG beads, respectively (Figure 2). These results indicate a significant difference, likely due to the freeze‒drying process and the formation of the respective alcogels. The metal contents of all the catalysts were determined via atomic absorption spectroscopy (AAS) (see Supplementary Table S1).

**FIGURE 3:**
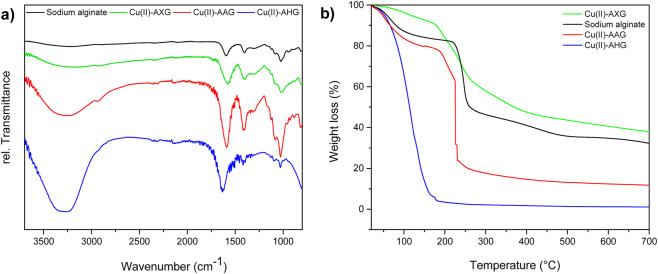
**(a)** Comparison of FT-IR spectra and **(b)** TGA results for all the materials. Sodium alginate (black line), Cu(II)-AXG beads (green line), Cu(II)-AAG beads (red line), and Cu(II)-AHG beads (blue line).

The FT-IR spectra of the Cu(II)-AHG beads revealed characteristic absorption bands at 1597 cm^−1^ and 1411 cm^−1^, corresponding to the asymmetric and symmetric stretching vibrations of the carboxylate functional groups, respectively (Figure 3a). Additionally, typical polysaccharide bands related to C–O, C–C, and C–O–H stretching vibrations were observed between 1176 cm^−1^ and 1028 cm^−1^. Overall, the incorporation of copper ions into the AHG beads did not significantly affect the main vibrational modes of the original sodium alginate. The broad bands between 3379 cm^−1^ and 3076 cm^−1^ were attributed to the stretching vibrations of the–OH functional groups. These bands were also present in the corresponding aerogels, although they appeared much less pronounced in the xerogels.

The water content was approximately 95%, as estimated by the following equation:

$$\%=\frac{\left[mass\left(hydrogel\right)-mass\left(xerogel\right)\right]}{mass\left(hydrogel\right)}\times 100$$

where the average weights were calculated from 20 beads.

The water content was confirmed via TGA. The analysis revealed a significant weight loss stage at approximately 100°C, which was attributed to the evaporation of water within the internal structure of the hydrogel. This weight loss accounts for approximately 95% of the total mass of the alginate beads, emphasizing the minimal polymer content required to form the hydrogel beads (Figure 3b, blue line). As shown in Figure 3b, the thermograms of the copper-alginate xerogel material and sodium alginate exhibited similar trends. The TGA thermogram of the xerogel material revealed a gradual weight loss below 100°C, which was attributed to the evaporation of adsorbed moisture and lactonization (Figure 3b, green line) (Martinsen et al., 1992). A major, rapid weight loss starting at approximately 200°C was associated with polymer decomposition through decarboxylation, whereas further weight loss above 280°C typically corresponds to the degradation of the alginate backbone and the cleavage of multiple hydroxyl groups (Achelhi et al., 2010). In contrast, the copper-alginate aerogel exhibited distinct thermal behavior. Approximately 20% of its weight was lost in the first stage (50°C–200°C) because of the evaporation of absorbed water. The second stage, accounting for approximately 75% of the total mass loss, occurred with a maximum at 240°C, which was attributed to the removal of the alginate polymer’s–OH side groups. This material did not fully degrade between 280°C and 700°C, retaining over 18% of its mass throughout this temperature range.

**FIGURE 4:**
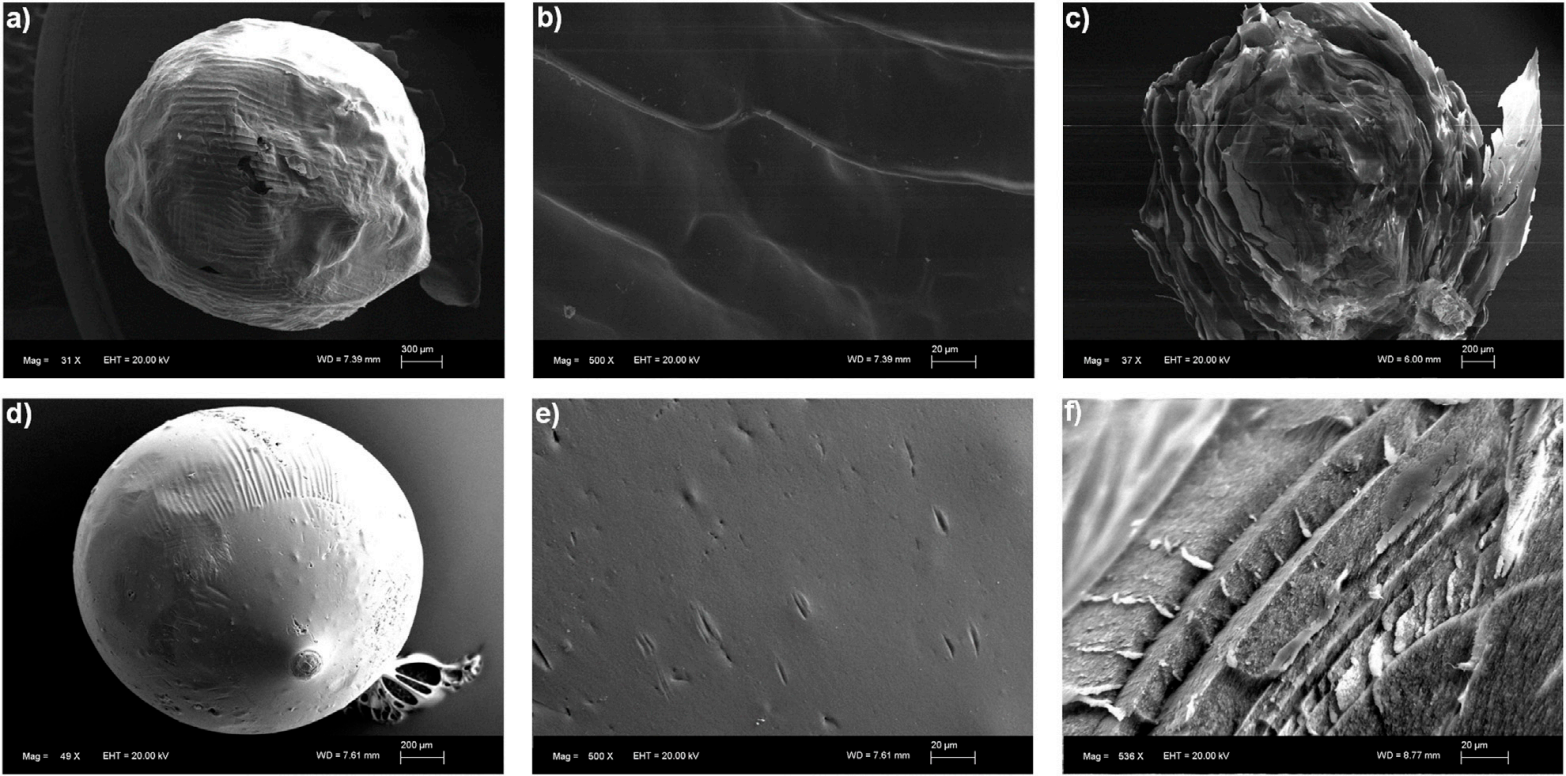
SEM micrographs of the Cu(II)-AX bead surface **(a,b)** and the Cu(II)-AX bead cross-section **(c)**. SEM micrographs of the Cu(II)-AA bead surface **(d,e)** and the Cu(II)-AA bead cross-section **(f)**.

The morphology of the Cu-alginate beads was analyzed by scanning electron microscopy, as shown in Figure 4. Both the xerogels and aerogels exhibited a spherical shape with rugged, dense surfaces that displayed some wrinkles and porosity. By increasing the magnification, layers with cavities in the cross-sections of the copper alginate were observed (Figures 4c,f).

**FIGURE 5:**
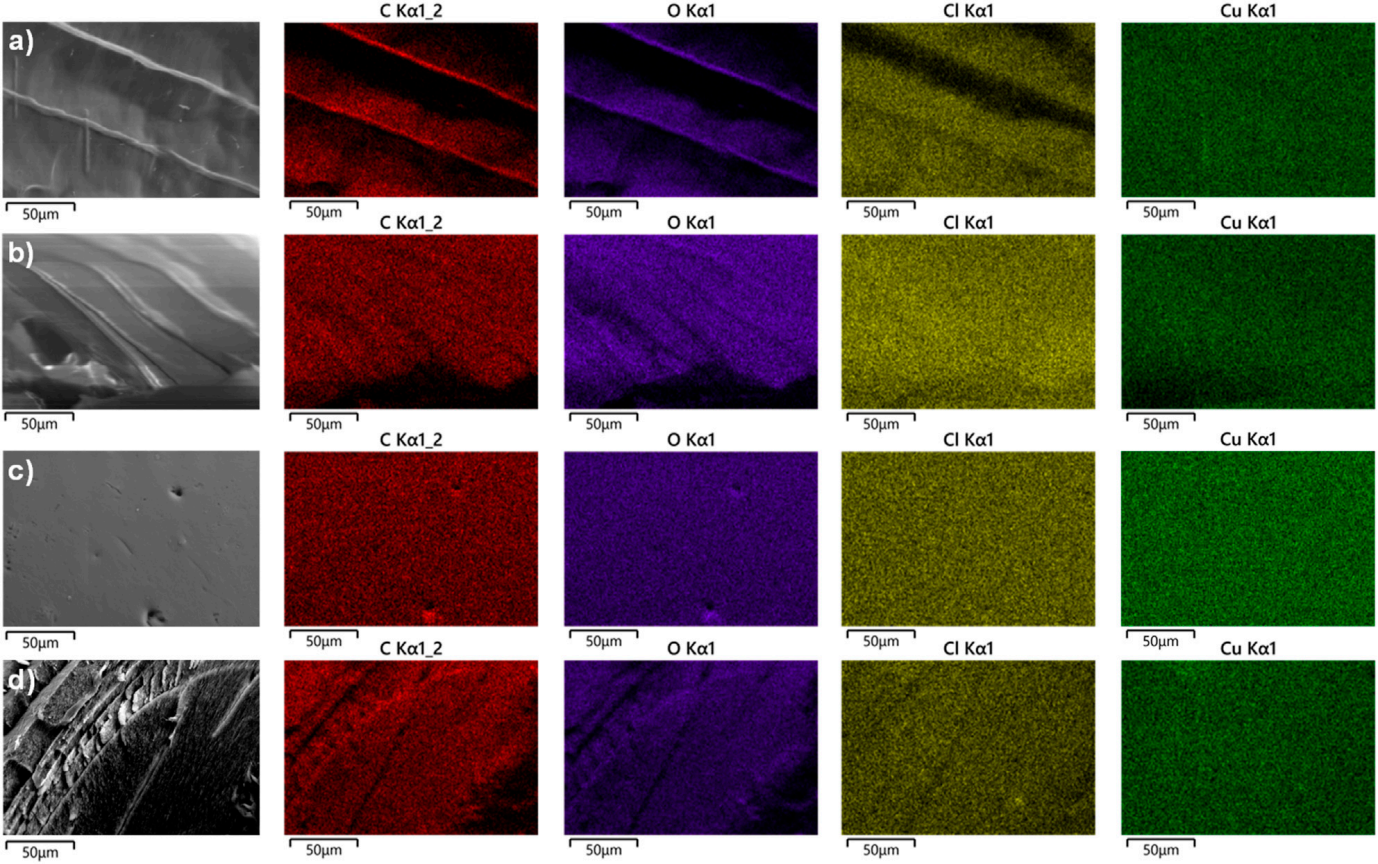
Energy-dispersive X-ray mapping images of the **(a)** Cu(II)-AXG surface; **(b)** Cu(II)-AXG cross-section; **(c)** Cu(II)-AAG surface; and **(d)** Cu(II)-AAG cross-section.

The chemical homogeneity of the Cu^2+^-crosslinked beads was analyzed via EDX (Figure 5). The mapping confirmed a homogeneous distribution of copper ions throughout the material. Table 1 shows the chemical compositions of the Cu(II)-alginate xerogel and aerogel beads, both from the surface and from a cross-section (see EDX spectrum, ESI). The surface of the xerogel and aerogel beads has a higher copper content than the interior of the beads.

**TABLE 1:** Chemical compositions of the Cu(II)-alginate xerogel and aerogel beads.

Biocatalyst	C %	O %	Cu %	Cl %
Cu(II)-AXG surface	39.29	37.38	18.62	4.72
Cu(II)-AXG cross-section	42.02	40.21	12.70	5.07
Cu(II)-AAG surface	39.66	40.26	16.42	3.66
Cu(II)-AAG cross-section	41.92	44.40	11.26	2.42

**FIGURE 6:**
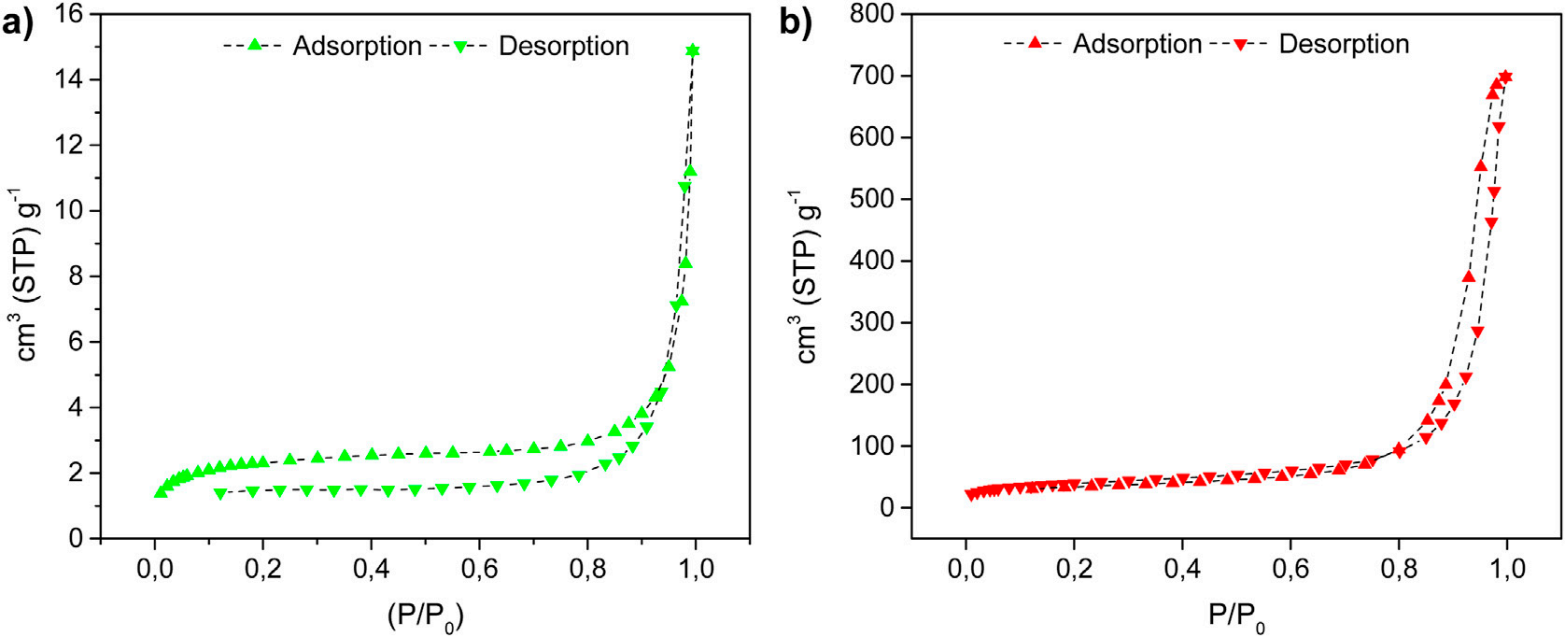
N_2_ sorption isotherms at 77 K for **(a)** Cu(II)-AXG (green) and **(b)** Cu(II)-AAG (red).

The textural properties of Cu(II)-crosslinked alginate beads are significantly influenced by the drying method. The aerogel has a high BET surface area (141.60 m^2^/g) and a substantial pore volume (0.7168 cm^3^/g), with an average pore diameter of 20.25 nm, confirming its mesoporous nature. In contrast, the xerogel has a much lower BET surface area (8.43 m^2^/g) and a reduced pore volume (0.0112 cm^3^/g), although it retains a mesoporous structure with an average pore diameter of 5.31 nm. Both materials display Type IV isotherms, which are typical of mesoporous materials, with hysteresis loops reflecting differences in pore uniformity: the aerogel has a narrow H1 loop, indicative of uniform pores, whereas the xerogel has a wider H2/H3 loop, which is associated with a less homogeneous pore structure (Figure 6).

The superior textural properties of the aerogel, including its greater surface area and pore volume, are attributed to the supercritical CO_2_ drying method, which preserves the porous structure by preventing pore collapse during solvent removal. In contrast, the xerogel, which was dried via lyophilization, exhibited a less developed porous network due to the formation of ice crystals during freezing, which can damage the pore structure. These differences highlight the critical role of the drying method in determining the final textural properties of the materials (Quignard et al., 2008).

### 3.2 Catalytic activity of Cu(II)-alginate beads in 1,3-dipolar cycloaddition between terminal alkynes and organic azides

Benzyl azide (**1a**) and phenylacetylene (**2a**) were selected as model substrates to evaluate the catalytic efficiency of various biocatalysts in the click reaction (Table 2). The reactions were conducted in water at room temperature. Notably, while benzyl azide (commercially available, ∼0.5 M in dichloromethane) achieves comparable product conversion after 24 h, a reduction in yield is observed at shorter reaction times. Therefore, all the experiments were conducted with solvent-free benzyl azide.

**TABLE 2:** Cu(II)-alginate beads catalyzed the 1,3-dipolar cycloaddition of benzyl azide and phenylacetylene^a^.

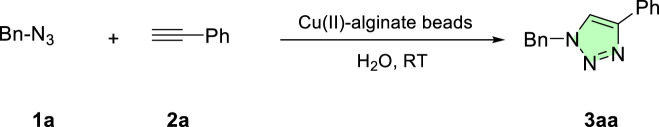				

Entry	Catalyst	Sodium ascorbate^[b]^	Time (h)	Yield %^[c]^
1	Cu(II)-AHG	-	24	0
2	Cu(II)-AXG	-	24	0
3	Cu(II)-AAG	-	24	0
4	Cu(II)-AHG	+	24	100
5	Cu(II)-AXG	+	24	100
6	Cu(II)-AAG	+	24	100
7	CuCl_2_·2H_2_O	-	24	<5
8	CuCl_2_·2H_2_O	+	24	57
9	-	+	24	0

^a^ Reaction conditions: **1a** (0.3 mmol), **2a** (0.3 mmol), biocatalyst or salt (2 mol%), sodium ascorbate (7 mol%), in H_2_O (2 mL) under air at room temperature.

^b^ [-] no sodium ascorbate added; [+] sodium ascorbate added.

^c^ Gas Chromatography (GC) yield.

As detailed in Table 2, no product formation was observed in the absence of a reducing agent (Entries 1–3). Since the click reaction (Kolb et al., 2001) is facilitated by copper(I) salts, we employed sodium ascorbate as a reducing agent to generate copper(I) *in situ* (Entries 4–6). With all the tested catalysts, complete conversion to the desired product was achieved after 24 h.

Furthermore, the reaction was performed with 2 mol% copper salt employed in the synthesis of the catalysts (Entries 7 and 8). As expected, no product was formed without sodium ascorbate. However, in the presence of the reducing agent, the triazole product was obtained in 57% yield after 24 h. Additionally, the reaction does not proceed in the absence of a copper catalyst (Entry 9).

Furthermore, the click reaction was performed using DMSO as the solvent instead of water, with sodium ascorbate at room temperature. The triazoles were obtained in yields of 20%, 43%, and 64% when the Cu(II)-AHG, Cu(II)-AXG, and Cu(II)-AAG catalysts were used, respectively. On the other hand, when the reaction was carried out in water and the reducing agent along with the reagents were added, the color of the beads changed from blue to light green, indicating the rapid formation of copper(I)-acetylide on their surface (see Supplementary Figure S1 in the ESI). In this context, it should be considered that although sodium ascorbate is soluble in water, it is poorly soluble in DMSO, which likely explains the low conversion observed when DMSO is used as solvent. This suggests that the formation of copper(I)-acetylide is limited by the low solubility of sodium ascorbate in the organic solvent. Additionally, the color change of the beads is less noticeable in this solvent.

**FIGURE 7:**
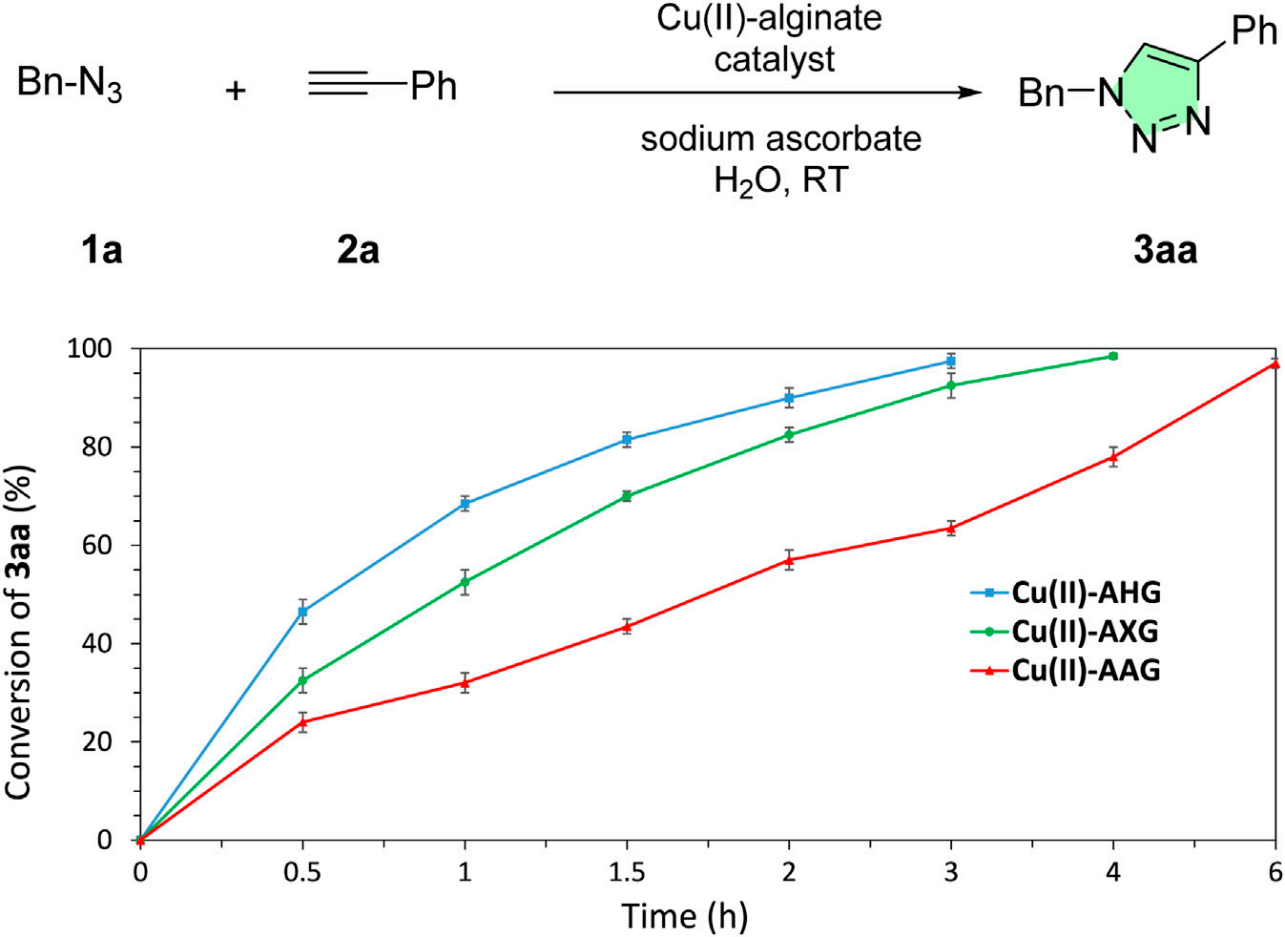
Kinetic profile for the click reaction promoted by Cu(II)-alginate beads. Reaction conditions: Benzyl azide (0.3 mmol), phenylacetylene (0.3 mmol), sodium ascorbate (1 mL, 7 mol%), Cu(II)-alginate beads (2 mol%), H_2_O (2 mL), at room temperature.

With these results in hand, we proceeded to investigate the reaction kinetics using the prepared Cu(II)-alginate catalysts (Figure 7).

As shown in Figure 7, the catalytic activity of the Cu(II)-AHG beads was notably superior to that of the corresponding aerogels and xerogels, driving the click reaction to completion within 3 h. On the basis of these results, we explored the scope of the reaction using the Cu(II)-AHG beads as catalysts in water and sodium ascorbate at room temperature (Table 3).

**TABLE 3:** 1,3-Dipolar cycloaddition between benzyl azide and different terminal alkynes catalyzed by Cu(II)-AHG beads.^[a]^

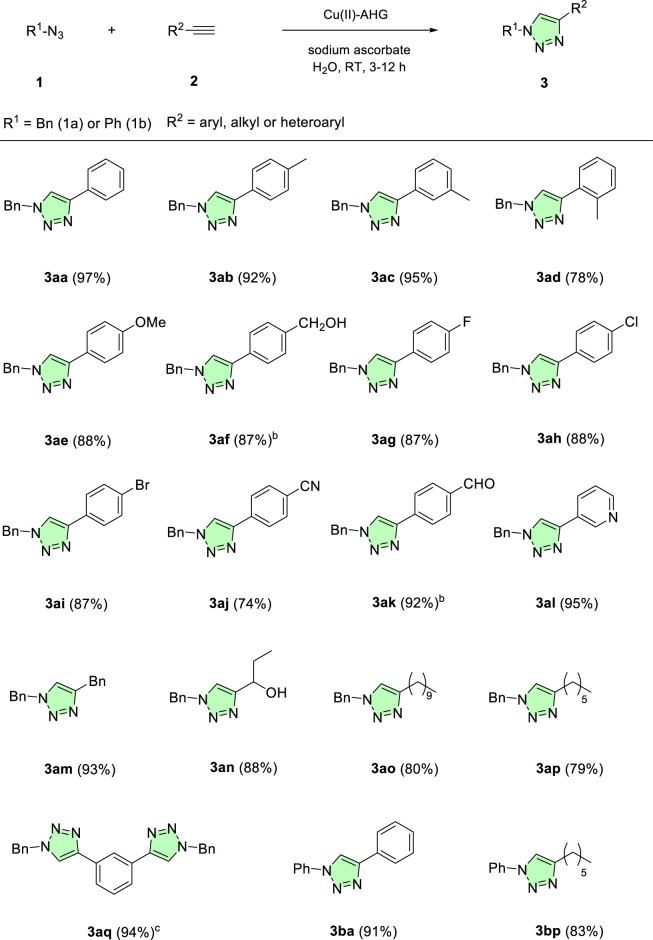

[a] Reaction conditions: Benzyl or phenyl azide (0.3 mmol), terminal alkyne (0.3 mmol), Cu(II)-AHG beads (5 beads, 2 mol%), sodium ascorbate (1 mL, 7 mol%), H_2_O (2 mL) at room temperature for 3–12 h. The percentage values correspond to the isolated yields. [b] The reaction was carried out in 12 h [c] Reaction conditions: benzyl azide (0.6 mmol), terminal alkyne (0.3 mmol), Cu(II)-AHG beads (10 beads, 2 mol%), sodium ascorbate (2 mL, 7 mol%), H_2_O (4 mL) at room temperature for 12 h. The percentage values correspond to the isolated yields.

As shown in Table 3, the methodology was successfully applied to the click reaction between benzylazide (**1a**) and a broad range of terminal alkynes, featuring both electron-donating groups (-Me, -OMe, and -CH_2_OH) (Table 3, **3ab**-**3af**) and electron-withdrawing groups (-F, -Cl, -Br, -CN, and -CHO) (Table 3, **3ag**-**3ak**) on the aromatic ring, resulting in the corresponding triazoles with excellent isolated yields. The synthesis of triazoles from terminal alkynes containing heteroaromatic rings (compound **3al**) or alkyl terminal alkynes (**3am, 3an, 3ao,** and **3ap**) was also achieved. As observed, no significant difference in reactivity was noted among the various terminal alkynes studied. Additionally, the methodology was successfully employed to synthesize a bis-triazole from 1,3-diethynylbenzene (**3aq**), although this compound required a longer reaction time. Finally, the protocol was applied to the synthesis of triazoles **3ba** and **3bp,** derived from phenyl azide (**1b**) and two different terminal alkynes, one aryl and one alkyl, demonstrating that the methodology is also compatible with different types of azides. Most of the products obtained were isolated through simple filtration or extraction with an organic solvent, without the need for further purification, making the employed methodology more sustainable and easier to implement.

In terms of recyclability, the Cu(II)-AHG beads were successfully reused up to three times with minimal loss of activity. However, gradual deactivation was observed after the third cycle (Table 4). The recycling process involved separating the beads from the reaction medium, transferring them to a new reaction vial, and adding fresh water, a reducing agent, and reactants. Consistent with previous studies, the observed decline in activity could be attributed to factors such as catalyst surface blockage by starting materials (Verrier et al., 2012), poisoning of the polysaccharide backbone (Chtchigrovsky et al., 2012), or textural instability (Valentin et al., 2005) under the reaction conditions.

**TABLE 4:** Recycling of the Cu(II)-AHG catalyst in 1,3-dipolar cycloaddition.

Cycles	Run 1	Run 2	Run 3	Run 4
Yield [%]^[^ ^a^ ^]^	98	90	82	60

^a^ Reaction conditions: Benzyl azide (0.3 mmol), phenylacetylene (0.3 mmol), Cu(II)-AHG (2 mol%, 5 beads), sodium ascorbate (1 mL, 7 mol%), H_2_O (2 mL) at room temperature. Isolated yield after 3 h.

**FIGURE 8:**
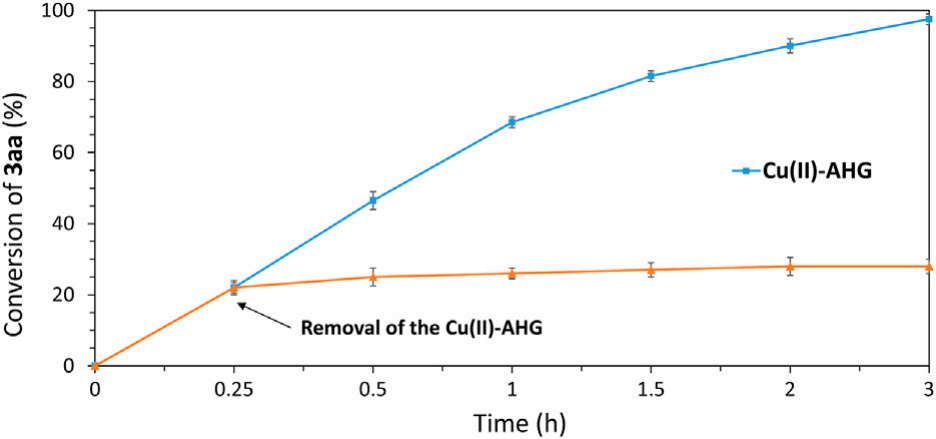
Control experiment showing the stagnation point of the reaction (blue squares) upon removal of the catalyst (orange triangle).

Control experiments additionally highlighted the heterogeneous nature of the process catalyzed by Cu^2+^-biohydrogels. To this end, the reaction of benzyl azide (**1a**) with phenylacetylene (**2a**) under standard conditions was stopped at 15 min of reaction time (22% conversion), and the catalyst was filtered off. The filtrate was then allowed to react for an additional 2 h, leading to a total conversion of 28%, as determined by GC analysis of the crude reaction mixture. As expected, stagnation of the reaction was observed after the removal of the catalyst (Figure 8).

Our catalyst exhibited a TON of 48.5 and a TOF of 16.7 h^−1^, demonstrating higher efficiency than other systems reported for CuAAC reactions using natural biopolymers as supports. (See Supplementary Table S2 in the ESI).

## 4 Conclusion

In summary, we report the preparation and characterization of copper ion-crosslinked alginate materials as highly efficient novel heterogeneous catalysts for azide‒alkyne cycloaddition (CuAAC) click chemistry in water at room temperature. The Cu(II)-alginate hydrogel beads demonstrated exceptional catalytic activity, outperforming xerogel and aerogel materials, even with low catalyst loading. This approach meets the sustainability requirements of green chemistry, offering an environmentally friendly and efficient solution for CuAAC reactions.

The protocol has proven to be more reliable than other reported methods, with various control experiments confirming that the use of a reducing agent is essential for successful reaction completion. The methodology was successfully applied to the synthesis of various triazoles with excellent yields, with most products not requiring purification. Furthermore, the catalyst was easily recovered by simple filtration and reused multiple times without significant loss of activity, making it a highly sustainable option. Importantly, the catalyst did not require any pretreatment for reuse, further supporting its green and sustainable nature.

Notably, while existing methods for similar catalysts often rely on complex syntheses or unsustainable components, our system prioritizes green chemistry principles: the alginate-based support is biodegradable, the reaction employs water as a solvent, and the process avoids energy-intensive conditions.

## Data Availability

The original contributions presented in the study are included in the article/Supplementary Material, further inquiries can be directed to the corresponding authors.
