# Correction: Critical assessment of copper-alginate hydrogel beads as recyclable and heterogeneous catalysts for aqueous azide‒alkyne cycloaddition

**DOI:** 10.3389/fchem.2025.1699753

**Published:** 2025-10-09

**Authors:** Yanina Moglie, Eduardo Buxaderas, Agoney González Cabrera, David Díaz Díaz

**Affiliations:** 1 Instituto de Química del Sur, INQUISUR (CONICET-UNS), Departamento de Química, Universidad Nacional del Sur, Bahía Blanca, Argentina; 2 AFM-NANO, Instituto Universitario de Bio-Orgánica Antonio González (IUBO-AG), Universidad de La Laguna, San Cristóbal de La Laguna, Spain; 3 Departamento de Química Orgánica, Universidad de La Laguna, San Cristóbal de La Laguna, Spain

**Keywords:** sustainable catalysis, copper alginate, click chemistry, 1,2,3-triazoles, green chemistry

Supplementary Data Sheet 1 was erroneously published with the original version of this paper. The file has now been replaced.

The original article has been updated.

