# Supplementary material for: Critical assessment of copper-alginate hydrogel beads as recyclable and heterogeneous catalysts for aqueous azide‒alkyne cycloaddition
Source: Front Chem. 2025 Aug 13;13:1644592. doi: 10.3389/fchem.2025.1644592 (PMC12380762; doi:10.3389/fchem.2025.1644592)
Supplement: Supplementary file 1 [file DataSheet1.pdf]

Supplementary Material

Critical assessment of the efficiency of copper-alginate hydrogels as recyclable and heterogeneous catalysts in azide–alkyne cycloadditions

Yanina Moglie,[1,2,3*] Eduardo Buxaderas,[1,2] Agoney González Cabrera,[2,3] David Díaz Díaz[2,3*]

[1]Instituto de Química del Sur, INQUISUR (CONICET-UNS), Departamento de Química, Universidad Nacional del Sur, Avda. Alem 1253, 8000 Bahía Blanca, Argentina. [2]AFM-NANO, Instituto Universitario de Bio-Orgánica Antonio González (IUBO-AG), Universidad de La Laguna, Avda. Astrofísico Francisco Sánchez 2, La Laguna 38206, Spain. [3]Departamento de Química Orgánica, Universidad de La Laguna, Avda. Astrofísico Francisco Sánchez 3, La Laguna 38206, Spain.

E-mail: ymoglie@ull.edu.es; ddiazdiaz@ull.edu.es

Table of Contents

1. Click reaction using Cu(II)-AHG beads S1

2. Metal loading S1

3. EDX Spectrum of xerogels and aerogels S2

4. TGA thermograms S3

5. Comparison with other catalysts S3

6. Characterization of compounds S5

7. RMN spectra data S8

8. References S24

1 Click reaction using Cu(II)-AHG beads

Figure S1 shows the visual appearance of the catalyst during the reaction progress.

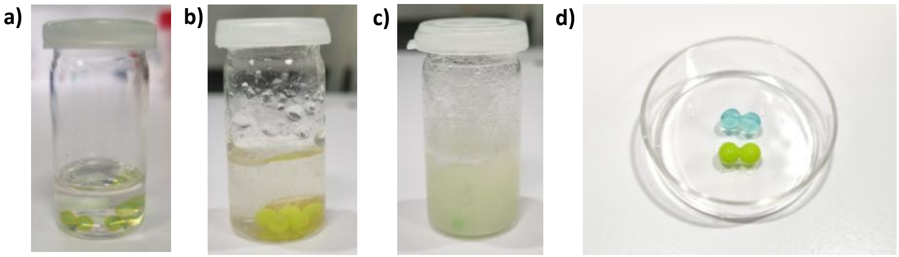

Figure S1. a) Reaction performed 10 minutes after initiation. b) Reaction performed 30 minutes after initiation. c) Reaction performed 3 hours after initiation. d) The blue beads represent fresh beads, while the yellow-green beads are the same as those in vial b).

2 Metal Loading

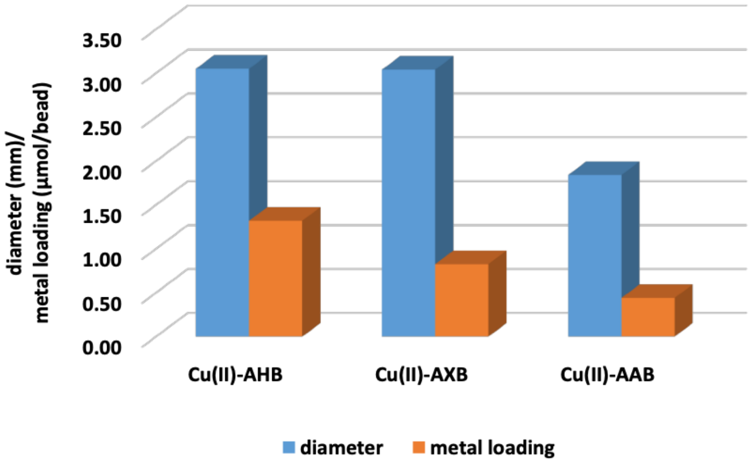

Figure S2. Correlation between the diameter of the Cu(II)-crosslinked alginate beads diameter (blue bars) and the corresponding metal loading (orange bars).

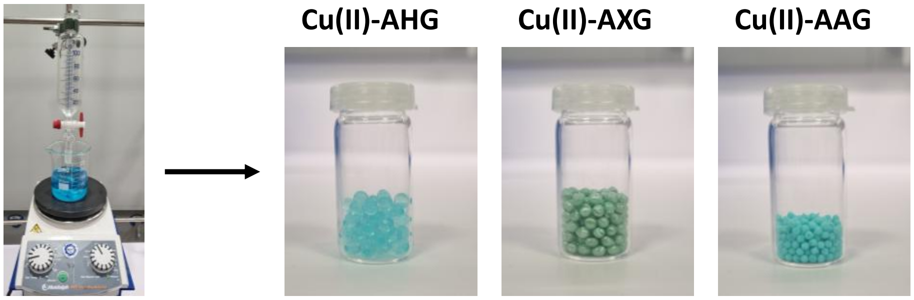

Figure S3. Visual appearance of copper alginate beads prepared as describe in Experimental Section.

Table S1. Correlation between the diameter of the Cu(II)-crosslinked alginate beads diameter and the corresponding metal loading.

Entry	Biocatalyst	Diameter (mm)	Metal loading (μmol/bead)
1	Cu(II)-AHG	3.05 ± 0.16	1.32
2	Cu(II)-AXG	3.04 ± 0.20	0.83
3	Cu(II)-AAG	1.84 ± 0.11	0.44

3 EDX spectrum

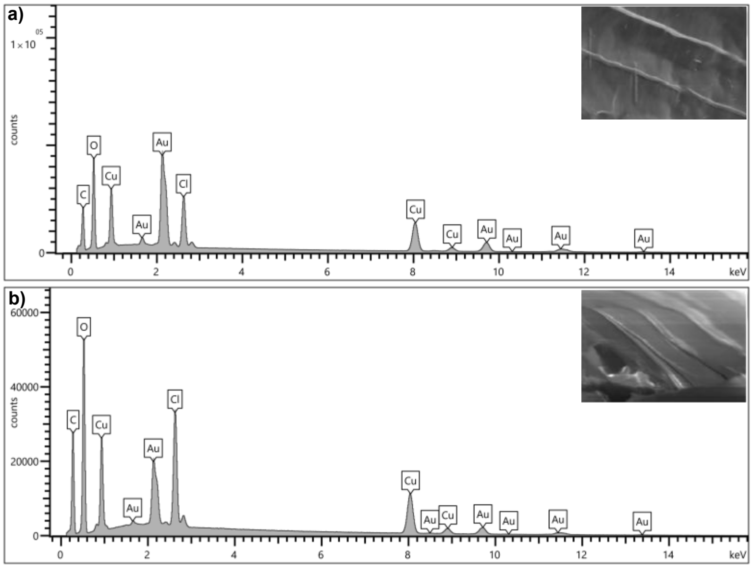

Figure S4. a) EDX spectrum of Cu(II)-AXG surface and b) Cu(II)-AXG cross-section.

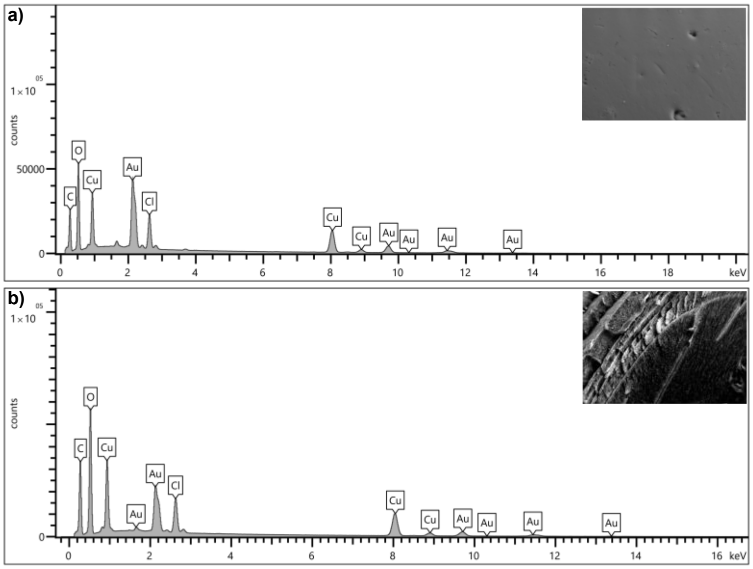

Figure S5. a) EDX spectrum of Cu(II)-AAG surface and b) Cu(II)-AAG cross-section.

4 TGA thermograms

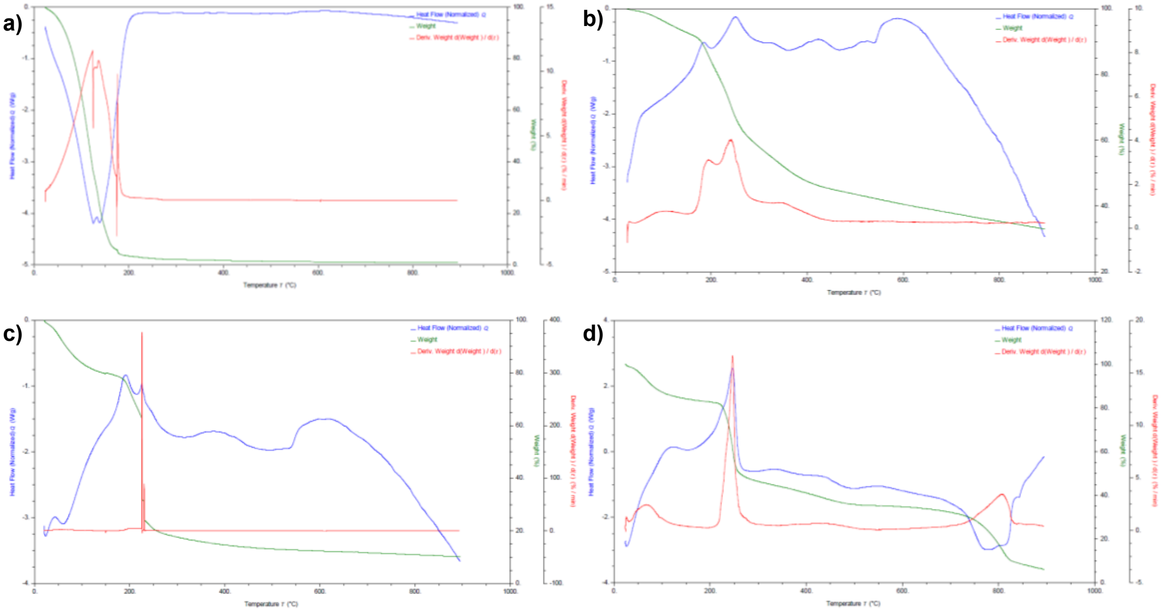

Figure S6. TGA curves of a) Cu(II)-AHG beads; b) Cu(II)-AXG beads; c) Cu(II)-AAG beads; d) sodium alginate.

5 Comparison with other catalysts

To highlight the efficiency of our catalytic system compared to other systems reported in the context of CuAAC, the comparative results for copper-catalyzed reactions, used as a model reaction, are summarized in Table S2.

As shown in the table, our catalyst demonstrated a high TON and TOF, outperforming most copper(II)-based catalysts supported on various biopolymers. Notably, the Cu(II)-loaded poly(hydroxamic acid) on cellulose catalyst exhibited excellent catalytic activity. However, its preparation involves multiple reaction steps related to cellulose modification, making it less sustainable. Additionally, the reaction requires high temperatures to proceed.[4] Similarly, the Cu(II)-cellulose nanofibers system also displayed remarkable catalytic performance, but its synthesis is labor-intensive, involving several reaction steps, which further reduces its sustainability.[6]

In contrast, our catalytic system not only combines high activity and simplicity in its preparation but also proves to be highly reliable and sustainable, offering a superior alternative to existing catalysts.

Table S2. Evaluation of the click synthesis of 1,4-disubstituted-1,2,3-triazoles via our protocols compared to other catalytic systems.

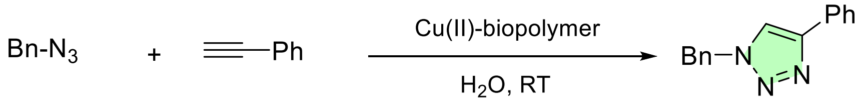

1a **2a** **3aa**

**Entry**	**Catalyst**	**Cu loading (mol%)**	**Conditions**	**Time (h)**	**Yield (%)**	**TON**	**TOF (h^{-1})**	**Ref.**
1	Cu(II)-AHG	2	H_2O, RT, SA[a] (7 mol%)	3	97	48.5	16.2	**This work**
2[b]	Cu(II)-alginate	2	H_2O, RT	24	95	24	1	Ref. 1
3	Cu(II)-alginate dried	2	H_2O, RT	48	93	23	0.48	Ref. 1
4	Cu(II)-alginate	21	H_2O, RT	18	98	4.6	0.26	Ref. 2
5	Cu(II)-cellulose	1.2	H_2O, RT	12	96	40	3.33	Ref. 3
6	Cu(II)-loaded poly(hydroxamic acid) on cellulose	0.05	H_2O, SA (5 mol%), 70 °C	2.5	96	1920	768	Ref. 4
7	Cu(II)-chitosan	n.d.[c]	H_2O, RT	4	99	n.d.[c]	-	Ref. 5
8	Cu(II)-cellulose nanofibers	3	H_2O, RT	8	88	55.5	6.93	Ref. 6

[a)]Sodium ascorbate. [b)]In our hands, this result could not be reproduced, yielding the corresponding triazole with a yield of less than 5%. [c)]Not determined.

6 Characterization of compounds

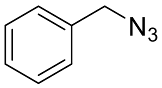

1-Benzylazide (1a):[7] yellow oil (98 %). ^{1}H NMR (500 MHz, $CDCl_3$): δ = 7.43–7.31 (m, 5H, Ar), 4.35 (s, 2H, CH_2) ppm. ^{13}C NMR (126 MHz, $CDCl_3$): δ = 135.5, 129.0, 128.4, 128.4, 128.3, 128.3, 55.0 ppm.

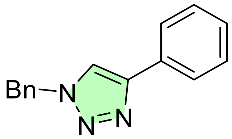

1-Benzyl-4-phenyl-1H-1,2,3-triazole (3aa):[7] white solid (97 %). ^{1}H NMR (500 MHz, $CDCl_3$): δ = 7.64–7.59 (m, 2H, Ar), 7.48 (s, 1H, CH), 7.24–7.17 (m, 5H, Ar), 7.15–7.10 (m, 3H, Ar), 5.57 (s, 2H, CH_2) ppm. ^{13}C NMR (126 MHz, $CDCl_3$): δ = 148.4, 134.8, 130. 7, 129.3, 128.9, 128.9, 128.3, 128.2, 125.8, 119.6, 54.4. ppm.

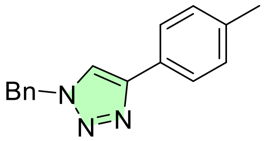

1-Benzyl-4-(*p*-tolyl)-1H-1,2,3-triazole (3ab):[8] pale yellow solid (92 %). ^{1}H NMR (500 MHz, $CDCl_3$): δ = 7.61–7.66 (m, 2H, Ar), 7.62 (s, 1H, CH), 7.41–7.35 (m, 3H, Ar), 7.33–7.29 (m, 2H, Ar), 7.23–7.17 (m, 2H, Ar), 5.57 (s, 2H, CH_2), 3.26 (s, 3H, CH_3) ppm. ^{13}C NMR (126 MHz, $CDCl_3$): δ = 148.5, 138.2, 134.9, 129.6, 129.3, 128.9, 128.2, 127.9, 125.8, 119.3, 54.4, 21.4 ppm.

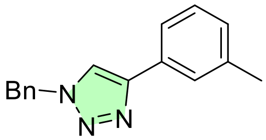

1-Benzyl-4-(*m*-tolyl)-1H-1,2,3-triazole (3ac):[9] white solid (95 %). ^{1}H NMR (500 MHz, $CDCl_3$): δ = 7.73–7.66 (m, 2H, Ar), 7.64–7.58 (m, 1H, Ar), 7.45–7.37 (m, 3H, Ar), 7.35–7.27 (m, 3H, Ar), 7.19–7.13 (m, 1H, Ar), 5.60 (s, 2H, CH_2), 2.40 (s, 3H, CH_3) ppm. ^{13}C NMR (126 MHz, $CDCl_3$): δ = 148.2, 138.7, 134.6, 130.1, 129.3, 129.2, 128.9, 128.8, 128.2, 126.6, 123.0, 119.8, 54.5, 21.5 ppm.

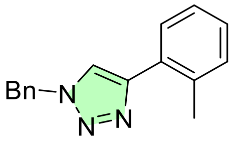

1-Benzyl-4-(*o*-tolyl)-1H-1,2,3-triazole (3ad):[10] white solid (78 %). ^{1}H NMR (500 MHz, $CDCl_3$): δ = δ 7.76–7.72 (m, 1H, Ar), 7.56 (s, 1H, Ar), 7.39–7.35 (m, 3H, Ar), 7.32–7.29 (m, 2H, Ar), 7.25–7.24 (m, 3H, Ar), 5.60 (s, 2H, CH_2), 2.42 (s, 3H, CH_3) ppm. ^{13}C NMR (500 MHz, $CDCl_3$) δ 147.6, 135.5, 134.8, 130.9, 129.9, 129.1, 128.9, 128.7, 128.2, 128.0, 126.1, 121.7, 54.2, 21.4 ppm.

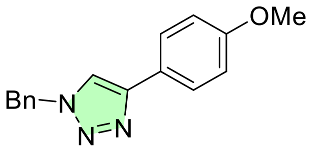

1-Benzyl-4-(4-methoxyphenyl)-1H-1,2,3-triazole (3ae):[9] yellow solid (88 %). ^{1}H NMR (500 MHz, $CDCl_3$): δ = 7.79–7.70 (m, 2H, Ar), 7.63 (s, 1H, CH), 7.42–7.34 (m, 3H, Ar), 7.33–7.28 (m, 2H, Ar), 6.96–6.90 (m, 2H, Ar), 5.55 (s, 2H, CH_2), 3.83 (s, 3H, CH_3) ppm. ^{13}C NMR (126 MHz, $CDCl_3$): δ = 160.2, 134.3, 129.4, 129.1, 128.4, 127.5, 122.0, 114.5, 55.5, 54.9 ppm.

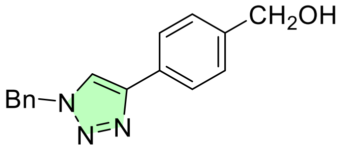

(4-(1-Benzyl-1H-1,2,3-triazol-4-yl)phenyl)methanol (3af):[11] white solid (87 %). ^{1}H NMR (500 MHz, $CDCl_3$): δ = 7.89–7.72 (m, 3H, Ar), 7.47–7.31 (m, 7H, Ar), 5.62 (s, 2H, CH_2), 5.31 (s, 1H, OH), 4.71 (s, 2H, CH_2) ppm. ^{13}C NMR (126 MHz, $CDCl_3$): δ = 147.4, 141.7, 134.3, 132.4, 129.4, 129.1, 128.4, 127.5, 126.2, 120.2, 65.0, 54.8 ppm.

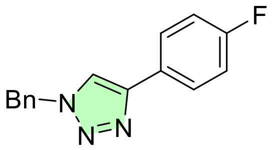

1-Benzyl-4-(4-fluorophenyl)-1H-1,2,3-triazole (3ag):[9] off white solid (87 %). ^{1}H NMR (500 MHz, $CDCl_3$): δ = 7.82–7.75 (m, 2 H, Ar), 7.67 (s, 1 H, CH), 7.43–7.35 (m, 3 H, Ar), 7.34–7.29 (m, 2 H, Ar), 7.09 (t, J = 8.3 Hz, 2H), 5.57 (s, 2 H, CH_2) ppm. ^{13}C NMR (126 MHz, $CDCl_3$): δ = 162.8 (d, J = 247.2 Hz), 134.7, 129.3, 129.0, 128.2, 127.6 (d, J = 8.1 Hz), 127.0, 115.9 (d, J = 21.8 Hz), 54.5 ppm.

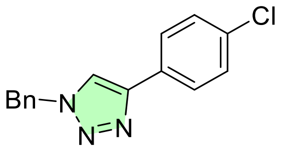

1-Benzyl-4-(4-chlorophenyl)-1H-1,2,3-triazole (3ah):[12] white solid (88 %). ^{1}H NMR (500 MHz, $CDCl_3$): δ = 7.73 (d, J = 8.30, 2H, Ar), 7.64 (s, 1 H, CH), 7.42–7.35 (m, 5 H, Ar), 7.34–7.30 (m, 2 H, Ar), 5.58 (s, 2 H, CH_2) ppm. ^{13}C NMR (126 MHz, $CDCl_3$): δ = 134.6, 134.0, 129.3, 129.2, 129.1, 129.0, 128.2, 127.1, 119.8, 54.5 ppm.

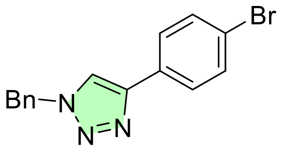

1-Benzyl-4-(4-bromophenyl)-1H-1,2,3-triazole (3ai):[13] white solid (87 %). ^{1}H NMR (500 MHz, $CDCl_3$): δ = 7.67 (d, J = 8.45, 2H, Ar), 7.65 (s, 1 H, CH), 7.52 (d, J = 8.53, 2H, Ar), 7.41–7.34 (m, 3 H, Ar), 7.32–7.27 (m, 2 H, Ar), 5.57 (s, 2 H, CH_2) ppm. ^{13}C NMR (126 MHz, $CDCl_3$): δ = 134.4, 132.1, 129.3, 129.2, 129.1, 128.3, 127.4, 122.4, 54.7 ppm.

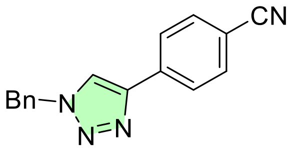

4-(1-Benzyl-1H-1,2,3-triazol-4-yl)benzonitrile (3aj):[14] white solid (92 %). ^{1}H NMR (500 MHz, $CDCl_3$): δ = 7.91 (d, *J* = 7.55 Hz, 2H), 7.74, (s, 1H), 7.69 (d, *J* = 7.46 Hz, 2H), 7.43–7.37 (m, 3H, Ar), 7.35–7.30 (m, 2H, Ar), 5.60 (s, 2H, CH_2) ppm. ^{13}C NMR (126 MHz, $CDCl_3$): δ = 146.4, 134.9, 134.2, 132.7, 129.3, 129.0, 128.2, 126.1, 120.6, 118.7, 111.5, 54.5 ppm.

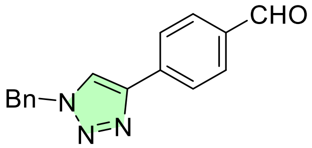

4-(1-Benzyl-1H-1,2,3-triazol-4-yl)benzaldehyde (3ak):[12] white solid (92 %). ^{1}H NMR (500 MHz, $CDCl_3$): δ = 10.01 (s, 1H, CHO), 7.98 (d, *J* = 7.54 Hz, 2H), 7.91 (d, *J* = 8.04 Hz, 2H), 7.79 (s, 1H, Ar), 7.45–7.29 (m, 5H, Ar), 5.63 (s, 2H, CH_2) ppm. ^{13}C NMR (126 MHz, $CDCl_3$): δ = 191.8, 147.0, 136.3, 136.0, 134.4, 130.5, 129.4, 129.2, 128.3, 126.2, 120.9, 54.6 ppm.

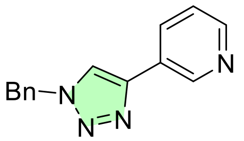

3-(1-Benzyl-1H-1,2,3-triazol-4-yl)pyridine (3al):[12] white solid (95 %). ^{1}H NMR (500 MHz, $CDCl_3$): δ = 8.98 (s, 1H, Ar), 8.55 (s, 1H, Ar), 8.24 (d, *J* = 6.36 Hz, 1H, Ar), 7.82 (s, 1H, Ar), 7.46–7.27 (m, 6H, Ar), 5.59 (s, 2H, CH_2) ppm. ^{13}C NMR (126 MHz, $CDCl_3$): δ = 148.4, 146.3, 144.9, 134.4, 133.8, 129.4, 129.1, 128.3, 127.3, 124.1, 120.2, 54.5 ppm.

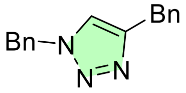

1,4-Dibenzyl-1H-1,2,3-triazole (3am):[15] pale brown solid (88 %). ^{1}H NMR (500 MHz, $CDCl_3$): δ = 7.30–7.24 (m, 3H, Ar), 7.23–7.11 (m, 7H, Ar), 7.09–7.04 (m, 1H, Ar), 5.39 (s, 2H, CH_2), 4.01 (s, 2H, CH_2) ppm. ^{13}C NMR (126 MHz, $CDCl_3$): δ = 147.9, 138.8, 134.6, 129.2, 128.9, 128.8, 128.8, 128.1, 126.7, 121.8, 54.4, 32.2 ppm.

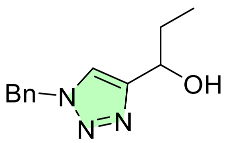

1-(1-Benzyl-1H-1,2,3-triazol-4-yl)propan-1-ol (3an): colorless oil (95 %). ^{1}H NMR (500 MHz, $CDCl_3$): δ = 7.43 (s, 1H, Ar), 7.40–7.33 (m, 3H, Ar), 7.29–7.23 (m, 2H, Ar), 5.47 (s, 2H, CH_2), 4.83–4.77 (m, 1H, CH), 3.58 (s, 1H, OH), 1.94–1.78 (m, 2H, CH_2), 0.95 (t, *J* = 7.42 Hz, 3H, CH_3) ppm. ^{13}C NMR (126 MHz, $CDCl_3$): δ = 151.9, 134.6, 129.2, 128.8, 128.2, 120.7, 68.3, 54.3, 30.3, 9.8 ppm. IR (neat, cm^{-1}): 3235, 2987, 2856, 1495, 1165, 1210, 1020, 788, 768 cm^{-1}. Elemental Analysis for $C_{12}H_{15}N_3O$: calcd. C 66.34; H 6.96; N 19.34; O 7.36; found. C 65.64; H 6.90; N 19.14; O 8.32.

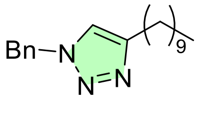

1-Benzyl-4-decyl-1H-1,2,3-triazole (3ao):[7] white solid (80 %). ^{1}H NMR (500 MHz, $CDCl_3$): δ = 7.35–7.27 (m, 3H, Ar), 7.25–7.16 (m, 3H, Ar), 5.46 (s, 2H), 2.71–2.62 (m, 2H, CH_2), 1.66–1.52 (m, 2H, CH_2), 1.31–1.10 (m, 14H, CH_2), 0.80 (t, J = 6.76, 3H, CH_3) ppm. ^{13}C NMR (126 MHz, $CDCl_3$): δ = 134.3, 129.3, 129.1, 128.3, 54.9, 32.0, 29.7, 29.6, 29.4, 29.4, 29.3, 29.3, 25.3, 22.8, 14.2 ppm.

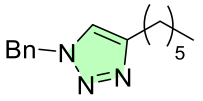

1-Benzyl-4-hexyl-1H-1,2,3-triazole (3ap):[7] white solid (79 %). ^{1}H NMR (500 MHz, $CDCl_3$): δ = 7.94 (s, 1H, Ar), 7.40–7.25 (m, 5 H, Ar), 5.56 (s, 2H, CH_2), 3.13–3.06 (d, J = 7.17 Hz, 2H, CH_2), 1.76–1.58 (m, 2H, CH_2), 1.37–1.26 (m, 6H, 3×CH_2), 0.89 (t, J = 6.6 Hz, 3 H, CH_3) ppm. ^{13}C NMR (126 MHz, $CDCl_3$): δ = 146.1, 131.5, 127.2, 127.1, 126.2, 123.1, 52.4, 37.4, 29.3, 21.5, 20.3, 11.8 ppm.

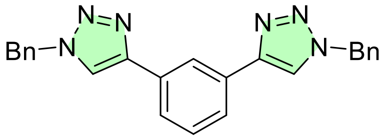

1,3-Bis(1-benzyl-1H-1,2,3-triazol-4-yl)benzene (3aq):[16] white solid (94 %). ^{1}H NMR (500 MHz, $CDCl_3$): δ = 8.21–8.19 (m, 1H, Ar), 7.79 (dd, J = 7.75, 1.70 Hz, 2H, Ar), 7.75 (s, 2H, Ar), 7.47–7.36 (m, 7H, Ar), 7.35–7.30 (m, 4H, Ar), 5.56 (s, 4H, CH_2) ppm. ^{13}C NMR (126 MHz, $CDCl_3$): δ = 147.9, 134.7, 131.2, 129.5, 129.3, 129.0, 128.3, 125.5, 123.0, 119.9, 54.4 ppm

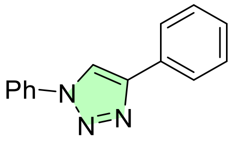

1-Phenyl-4-phenyl-1H-1,2,3-triazole (3ba):[7] white solid (91 %). ^{1}H NMR (500 MHz, $CDCl_3$): δ = 8.20 (s, 1 H, CH), 7.91 (d, J = 6.0 Hz, 2H, Ar), 7.80 (d, J = 6.2 Hz, 2H, Ar), 7.56 (t, J = 5.8 Hz, 2 H, Ar), 7.50–7.43 (m, 3 H, Ar), 7.40–7.34 (m, 1 H, Ar) ppm. ^{13}C NMR (126 MHz, $CDCl_3$): δ = 148.4, 137.1, 130.3, 129.8, 128.9, 128.8, 128.4, 125.9, 120.6, 117.6 ppm.

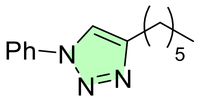

1-Phenyl-4-hexyl-1H-1,2,3-triazole (3bp):[17] white solid (83 %). ^{1}H NMR (500 MHz, $CDCl_3$): δ = 7.75–7.69 (m, 3H, Ar), 7.54–7.46 (m, 2H, Ar), 7.44–7.37 (m, 1H, Ar), 2.84–2.75 (m, 2H, CH_2), 1.79–1.66 (m, 2H, CH_2), 1.46–1.28 (m, 6H, 3 × CH_2), 0.95–0.86 (m, 3H, CH_3) ppm. ^{13}C NMR (126 MHz, $CDCl_3$): δ = 149.2, 137.3, 129.6, 128.4, 120.4, 118.8, 31.6, 29.4, 28.9, 25.7, 22.6, 14.1 ppm.

7 NMR spectra data

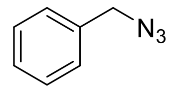

1a

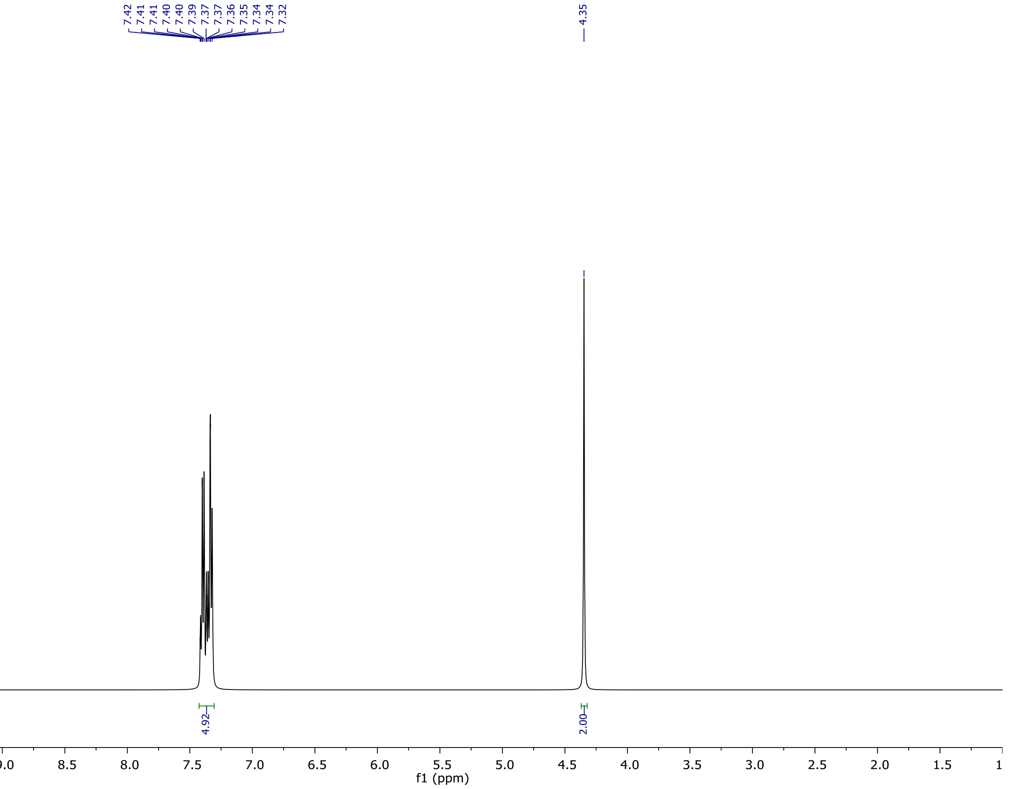

^{1}H NMR of benzylazide (1a)

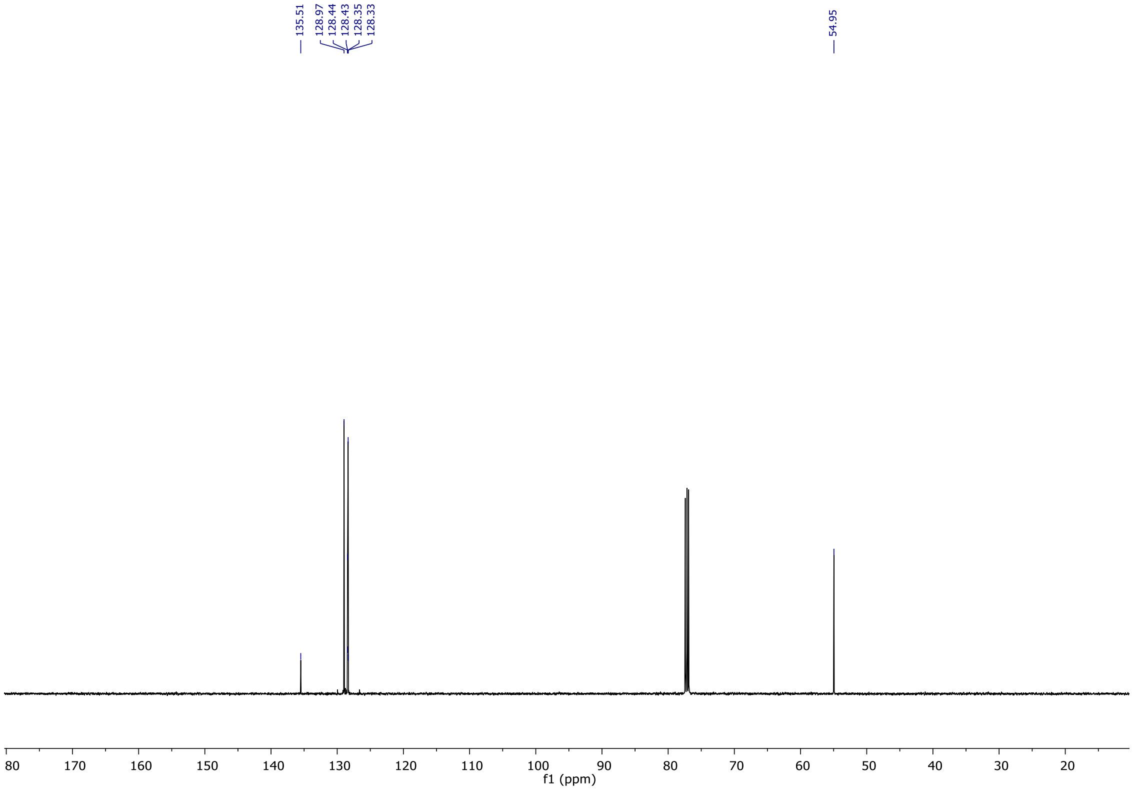

^{13}C NMR of benzylazide (1a)

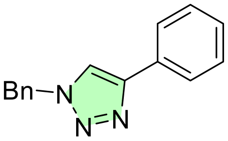

3aa

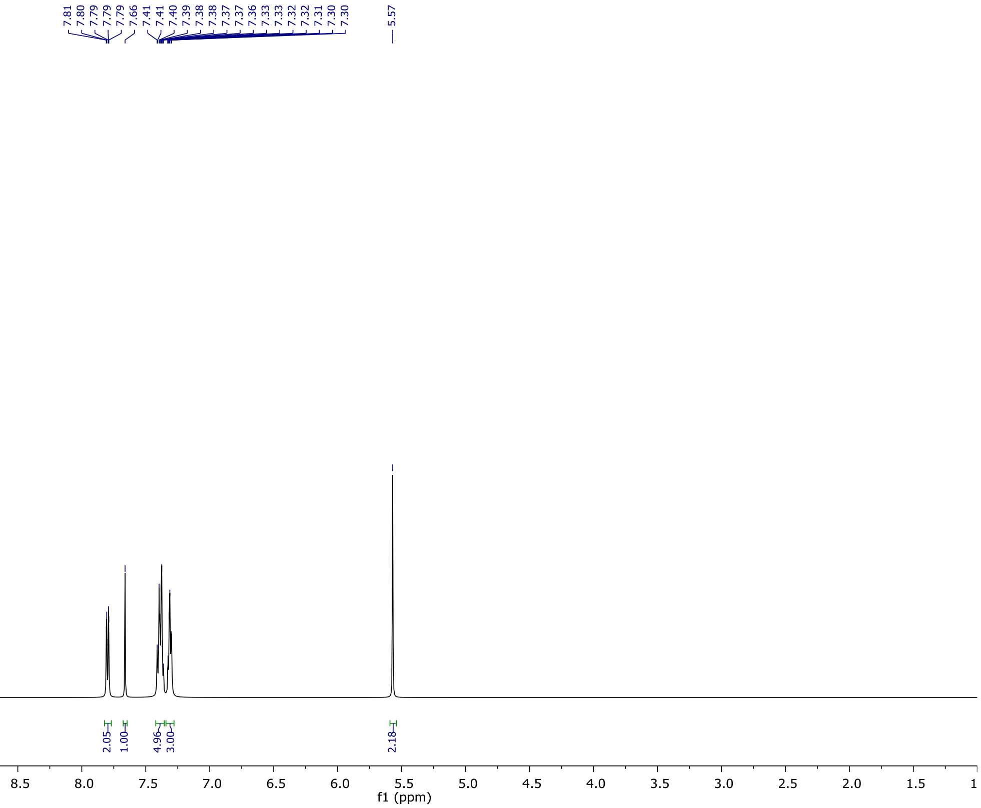

^{1}H NMR of 1-Benzyl-4-phenyl-1H-1,2,3-triazole (3aa)

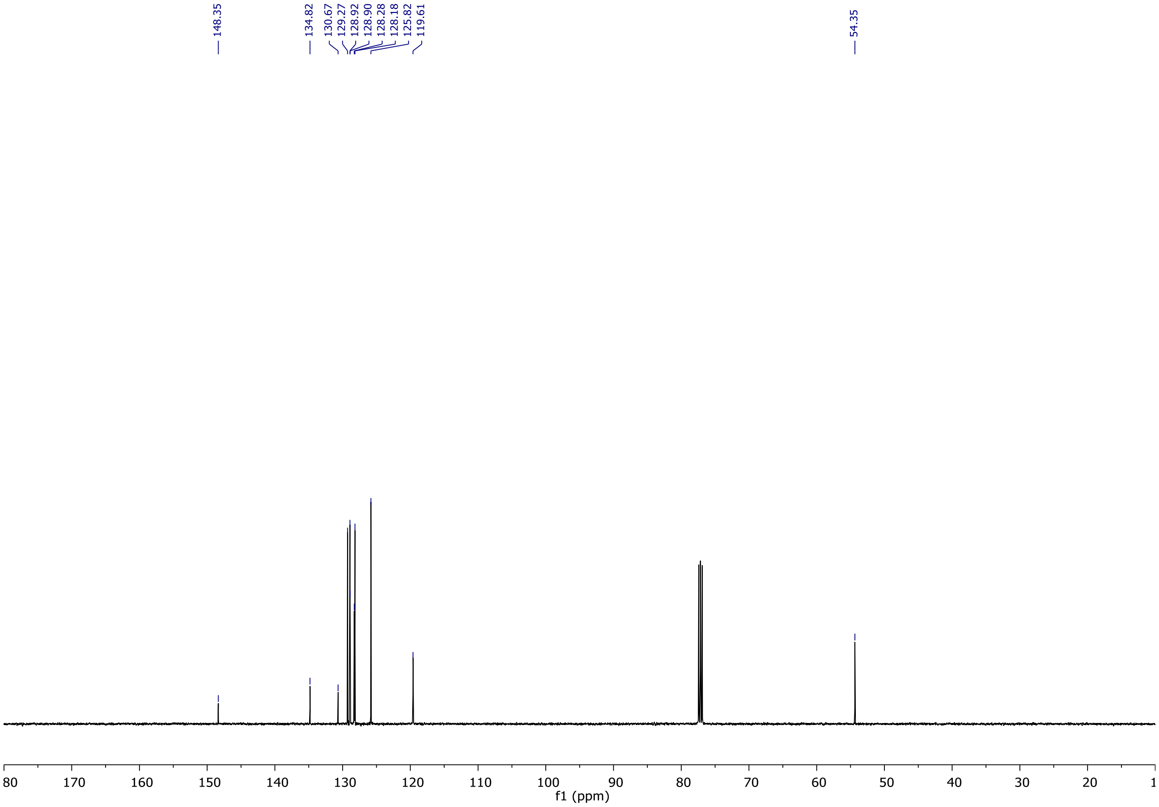

^{13}C NMR of 1-Benzyl-4-phenyl-1H-1,2,3-triazole (3aa)

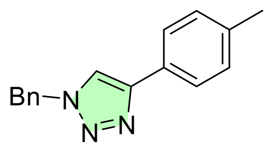

3ab

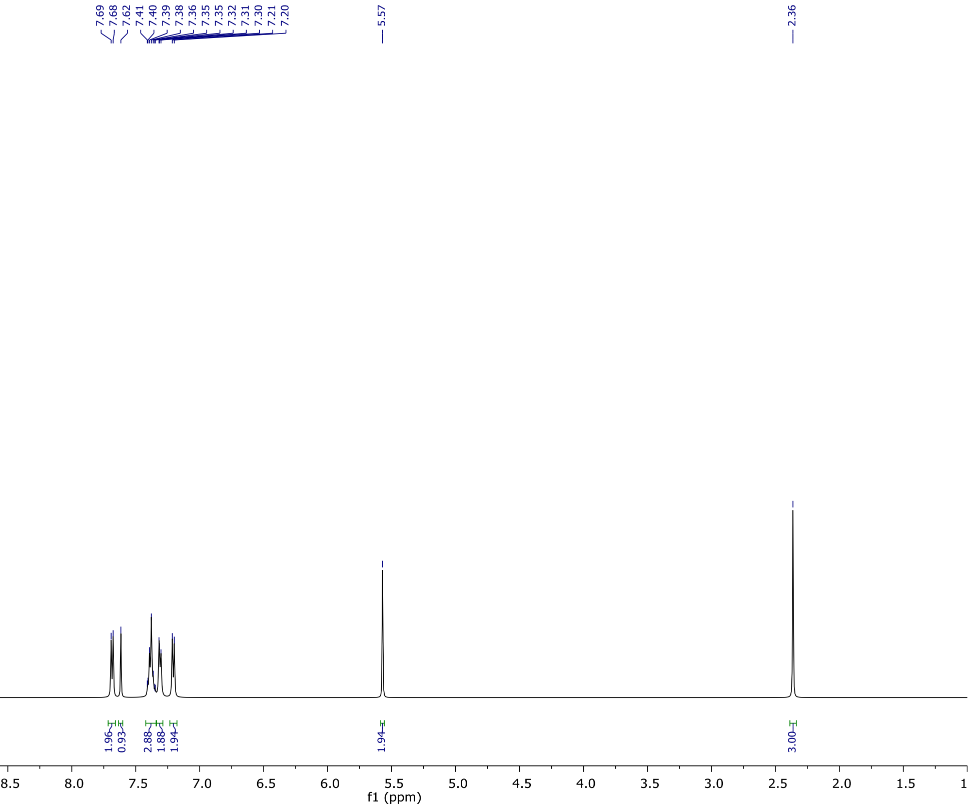

^{1}H NMR of 1-Benzyl-4-(*p*-tolyl)-1H-1,2,3-triazole (3ab)

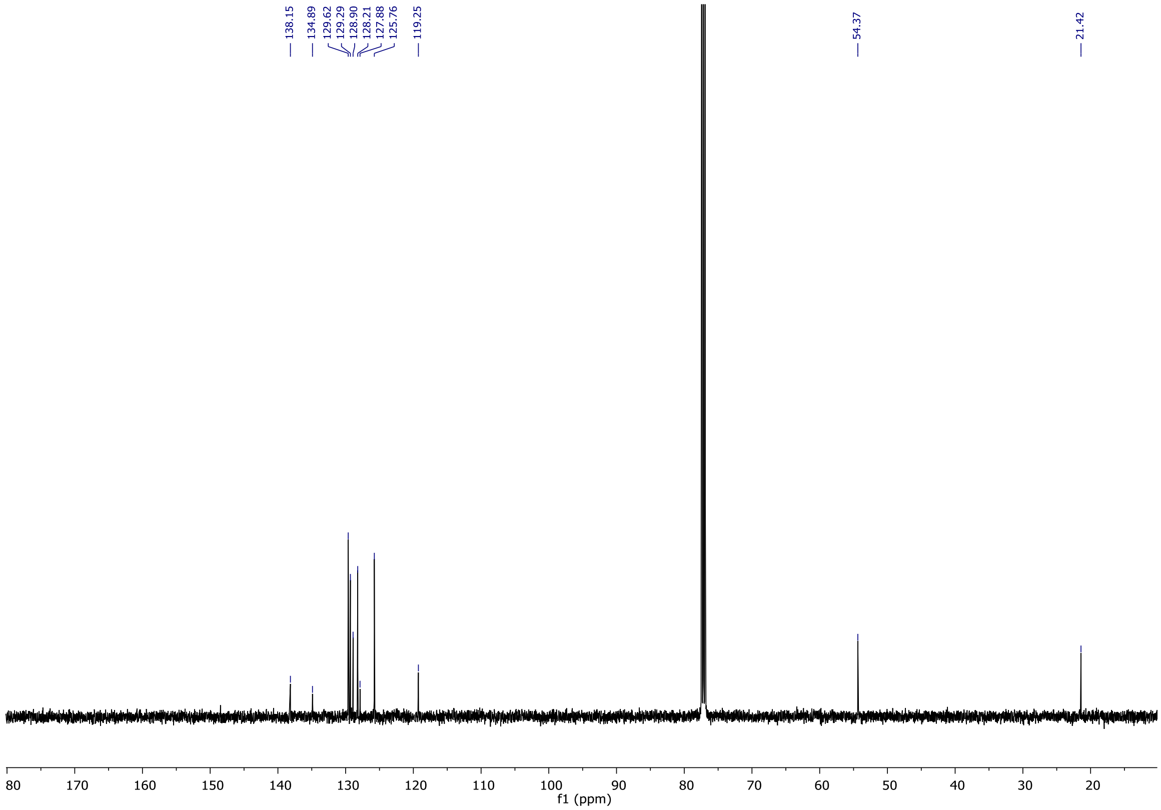

^{13}C NMR of 1-Benzyl-4-(*p*-tolyl)-1H-1,2,3-triazole (3ab)

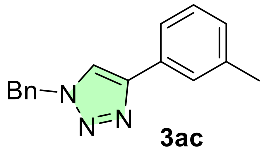

3ac

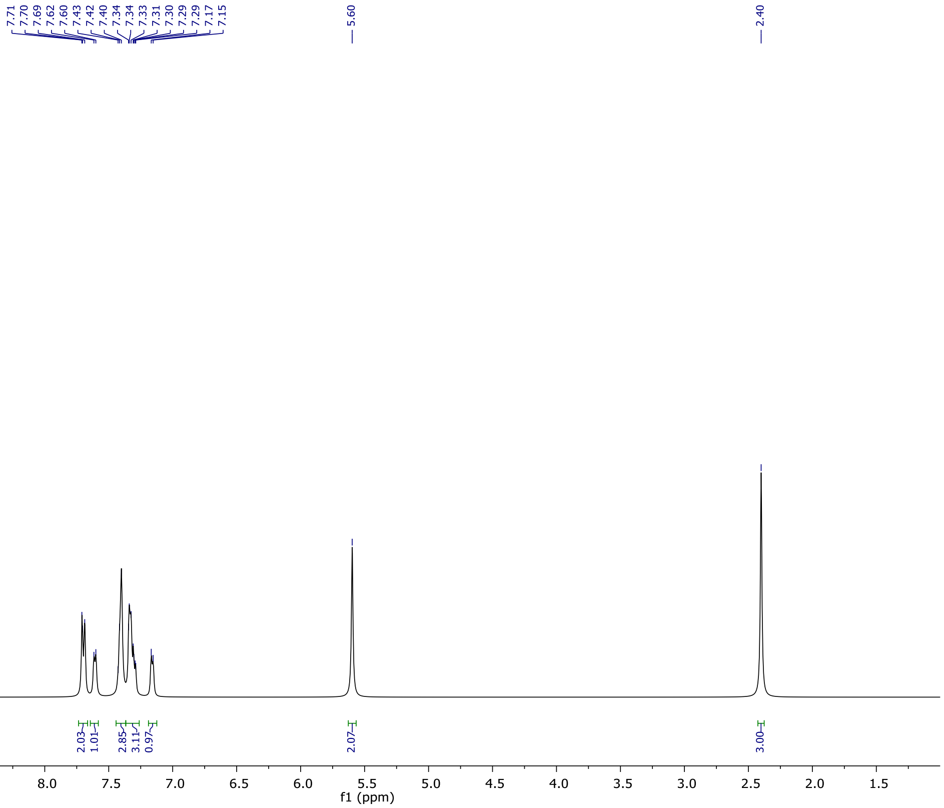

^{1}H NMR of 1-Benzyl-4-(*m*-tolyl)-1H-1,2,3-triazole (3ac)

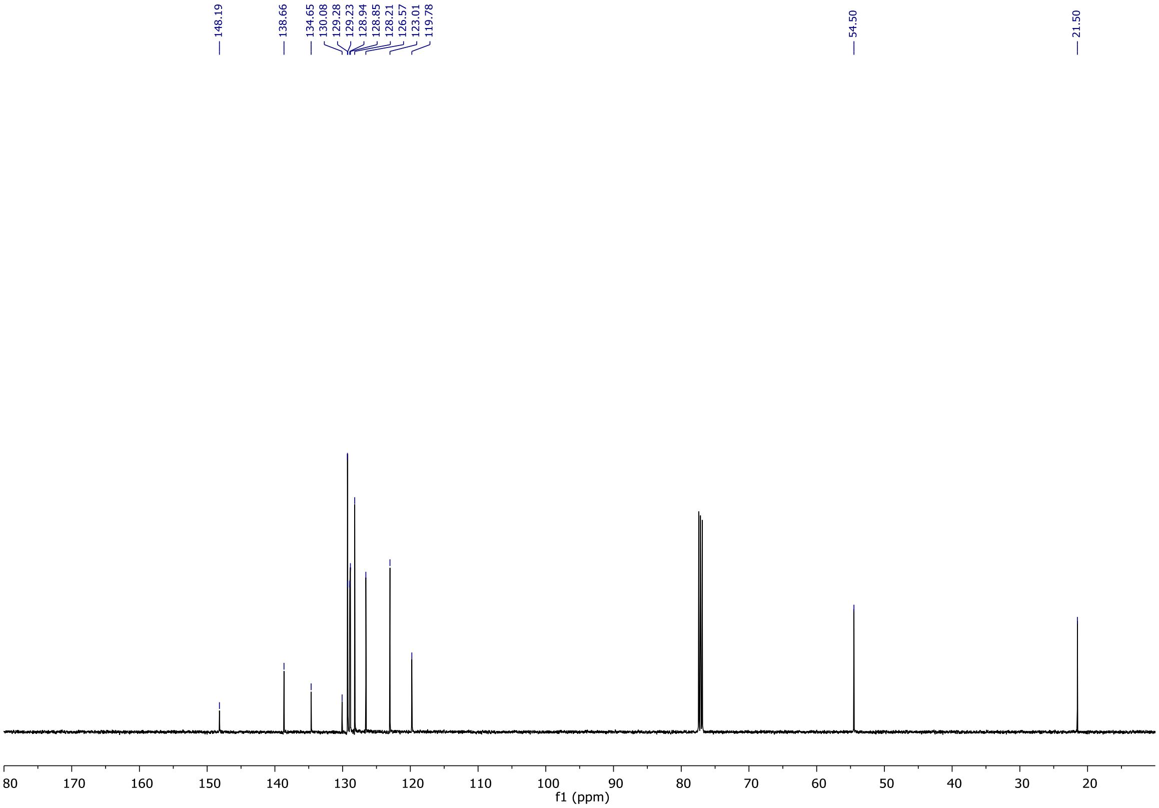

^{13}C NMR of 1-Benzyl-4-(*m*-tolyl)-1H-1,2,3-triazole (3ac)

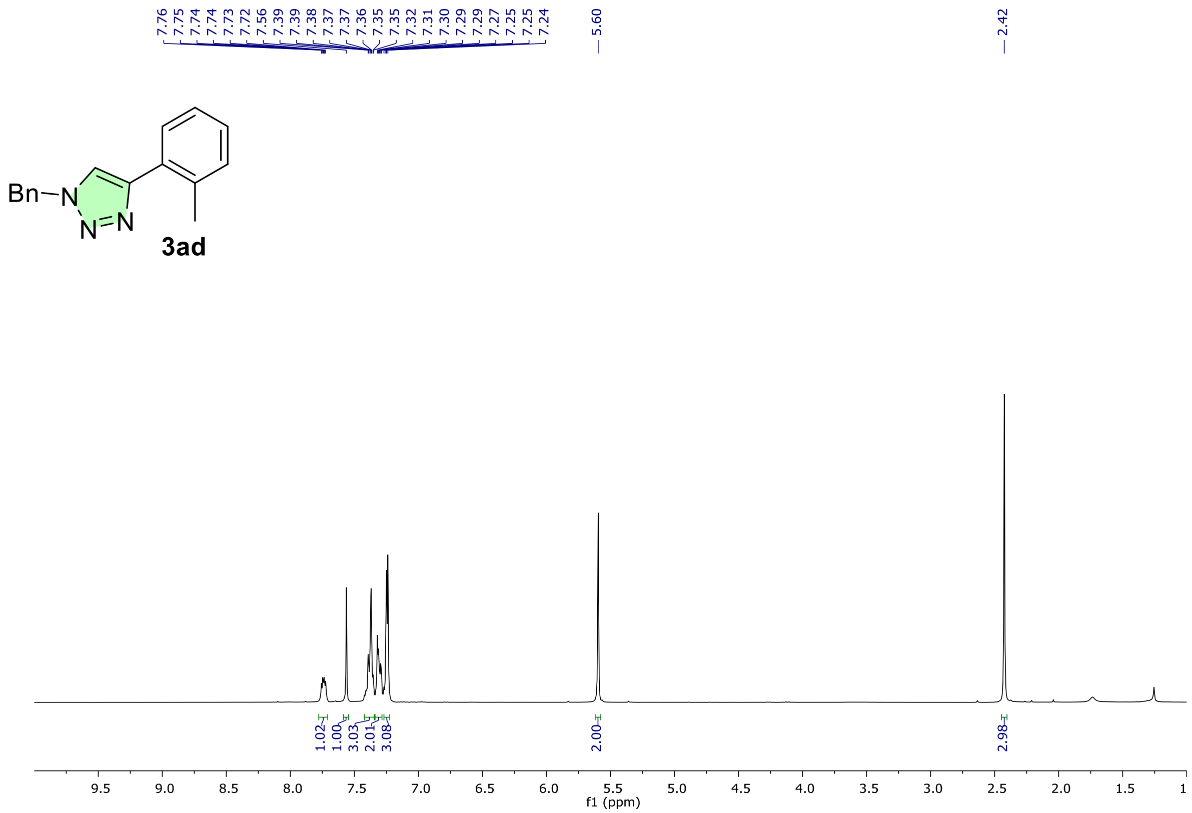

^{1}H NMR of 1-Benzyl-4-(*o*-tolyl)-1H-1,2,3-triazole (3ad)

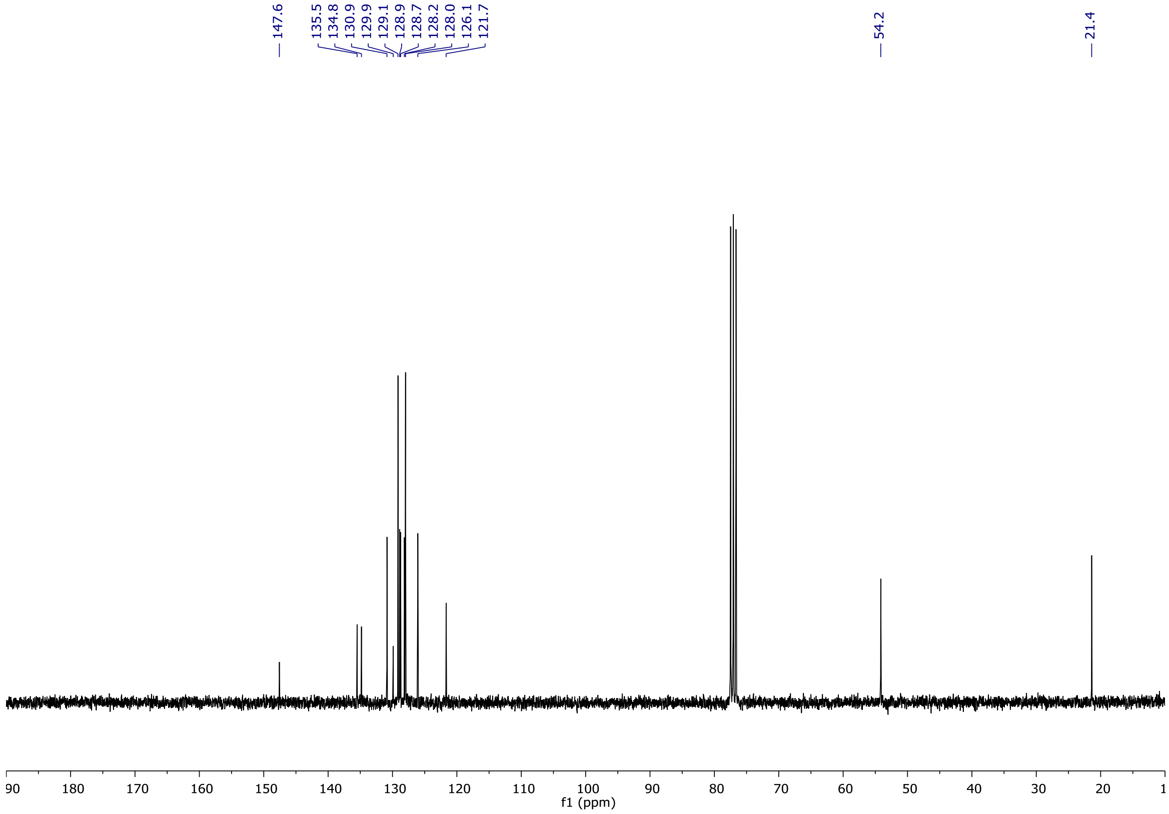

^{13}C NMR of 1-Benzyl-4-(*o*-tolyl)-1H-1,2,3-triazole (3ad)

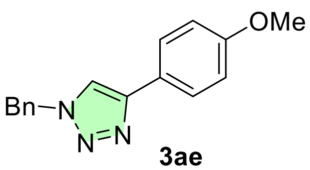

3ae

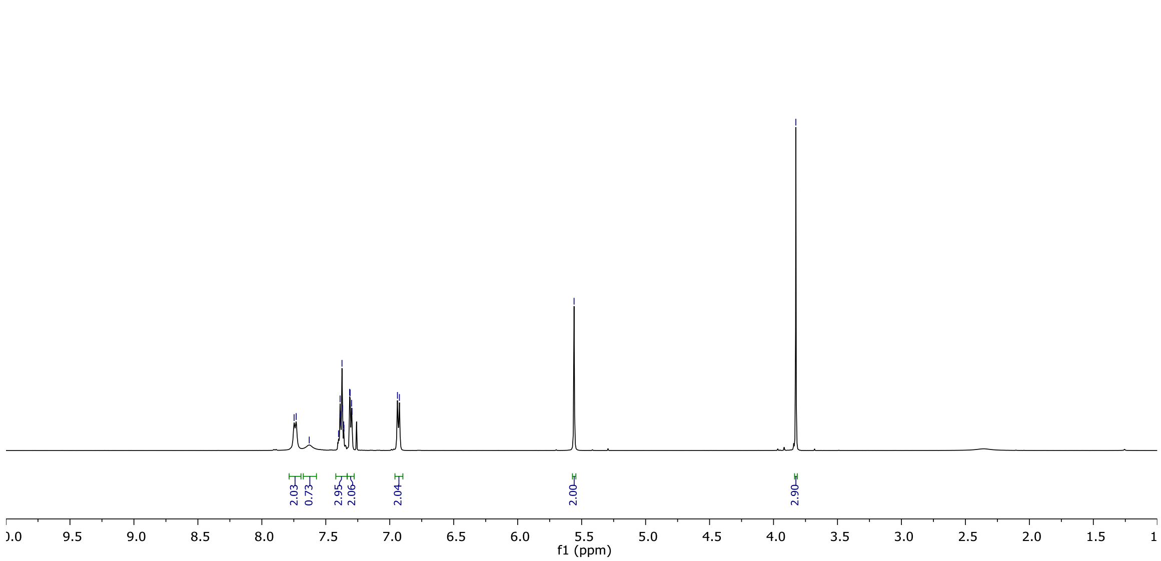

^{1}H NMR of 1-Benzyl-4-(4-methoxyphenyl)-1H-1,2,3-triazole (3ae)

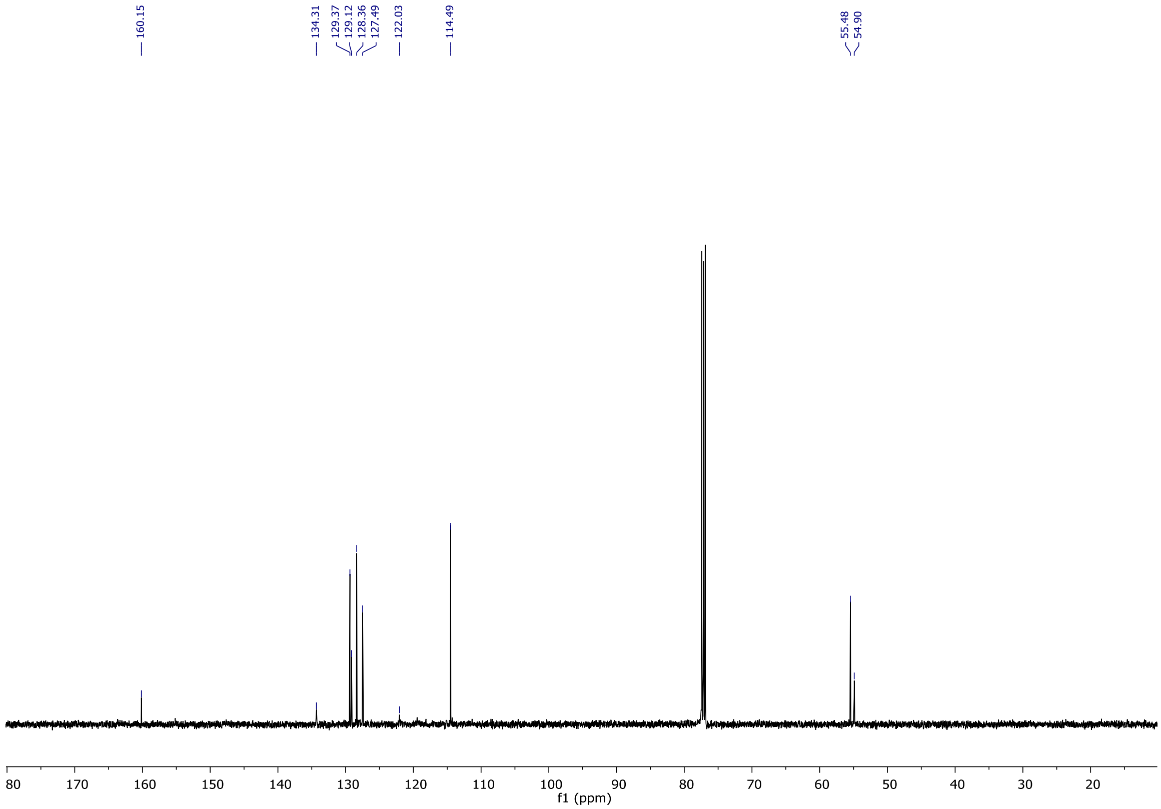

^{13}C NMR of 1-Benzyl-4-(4-methoxyphenyl)-1H-1,2,3-triazole (3ae)

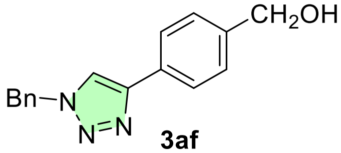

3af

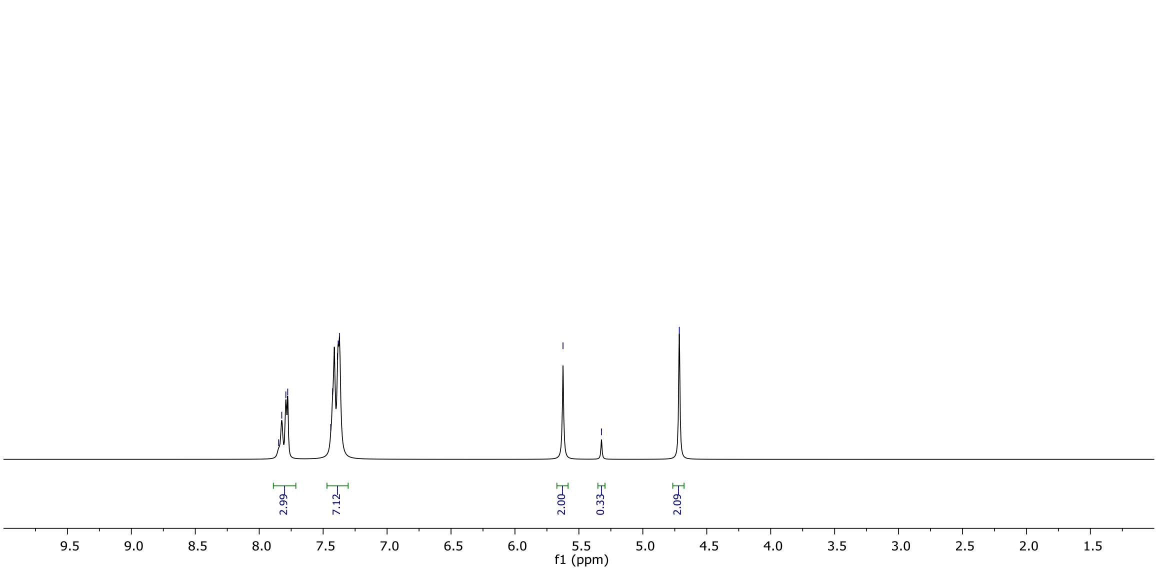

^{1}H NMR of (4-(1-Benzyl-1H-1,2,3-triazol-4-yl)phenyl)methanol (3af)

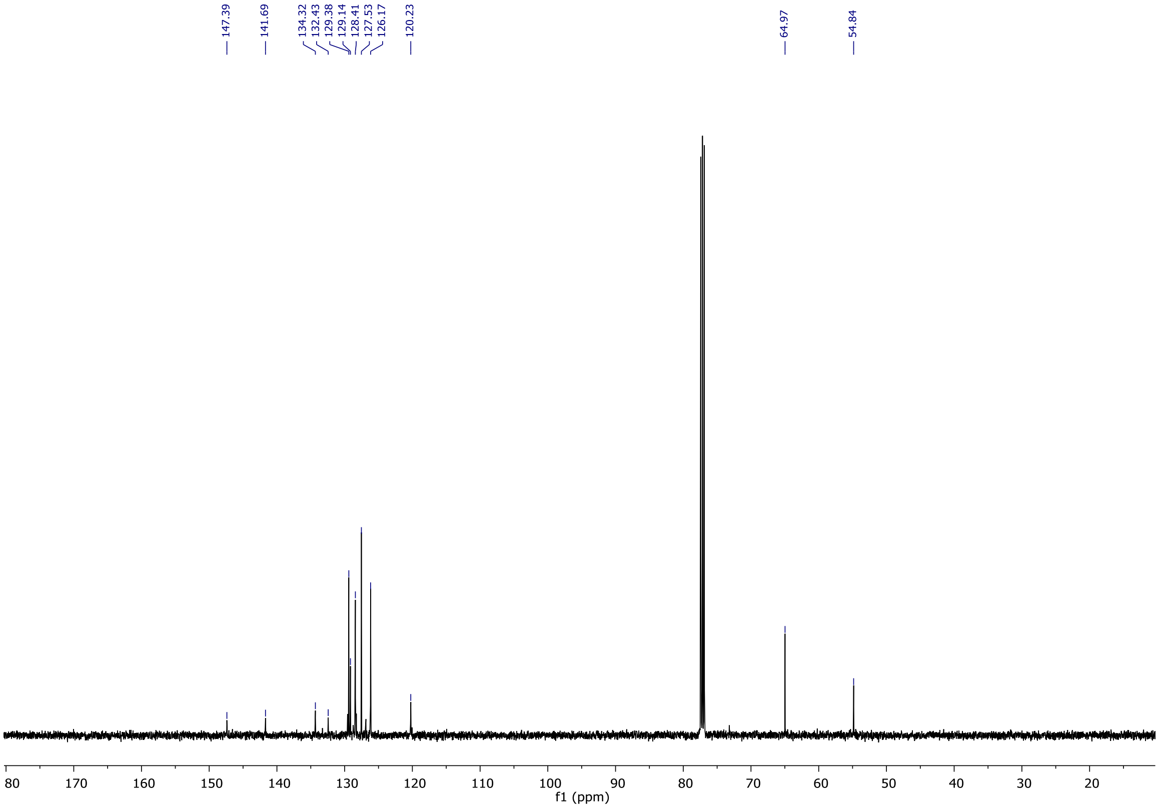

^{13}C NMR of (4-(1-Benzyl-1H-1,2,3-triazol-4-yl)phenyl)methanol (3af)

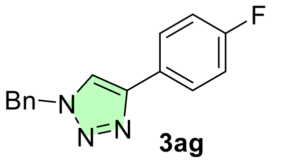

3ag

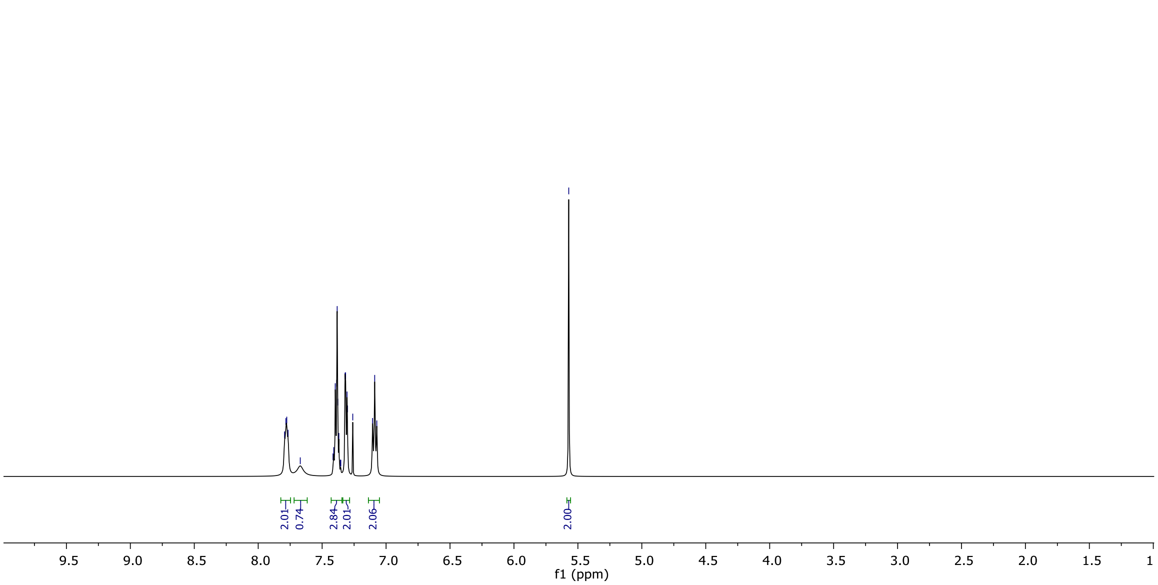

^{1}H NMR of 1-Benzyl-4-(4-fluorophenyl)-1H-1,2,3-triazole (3ag)

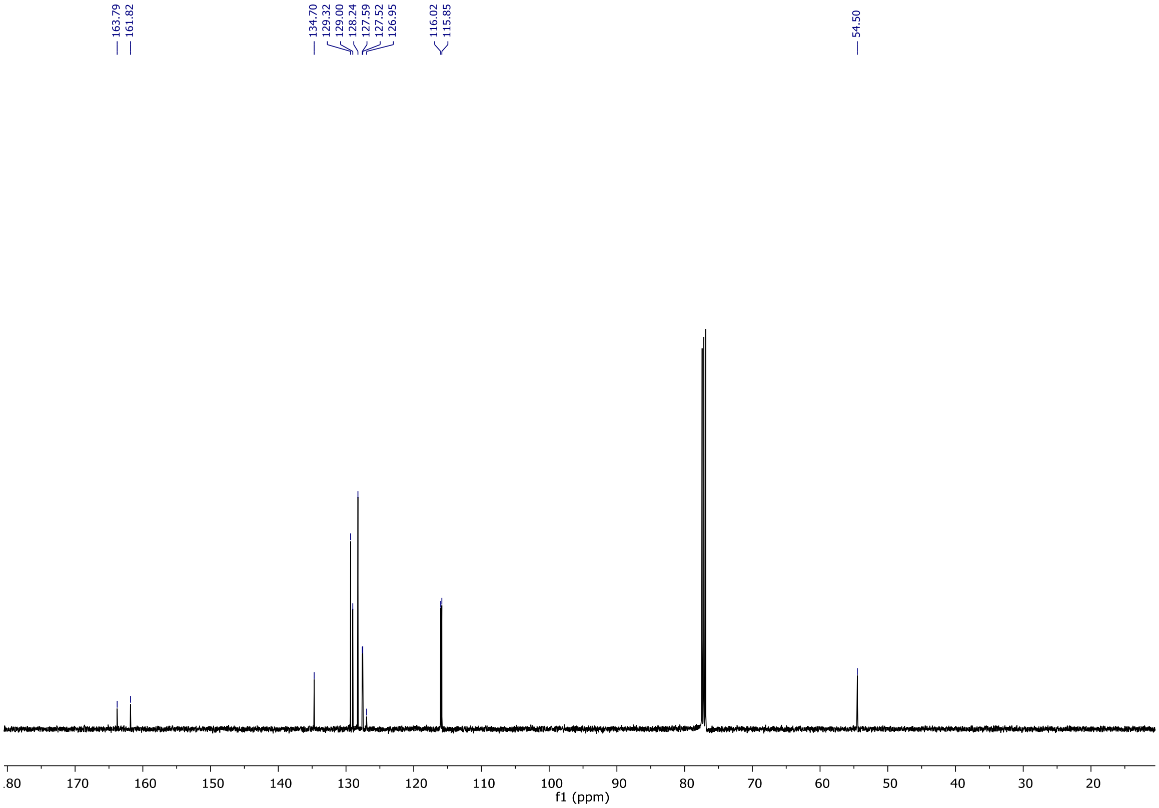

^{13}C NMR of 1-Benzyl-4-(4-fluorophenyl)-1H-1,2,3-triazole (3ag)

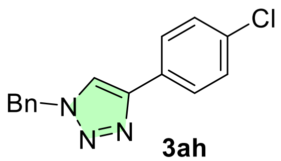

3ah

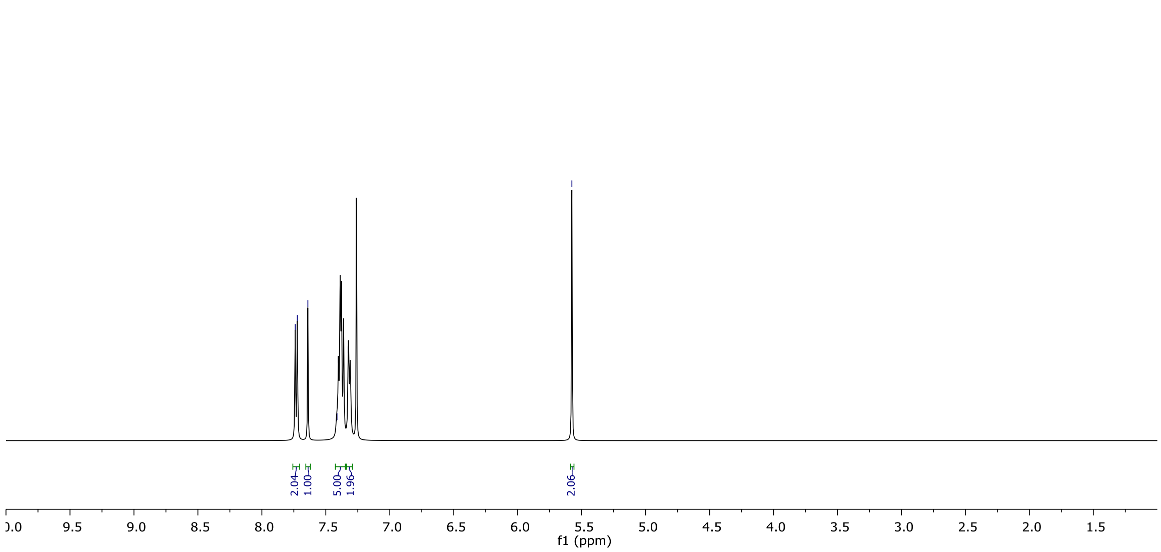

^{1}H NMR of 1-Benzyl-4-(4-chlorophenyl)-1H-1,2,3-triazole (3ah)

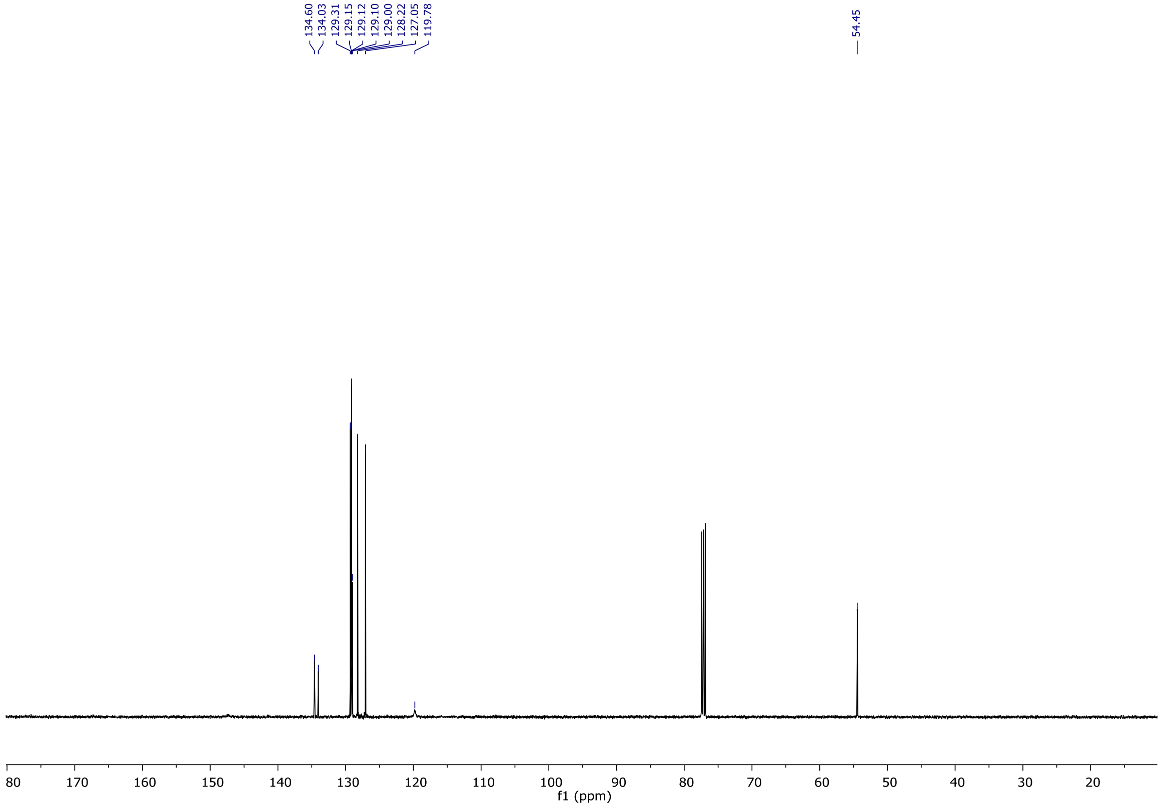

^{13}C NMR of 1-Benzyl-4-(4-chlorophenyl)-1H-1,2,3-triazole (3ah)

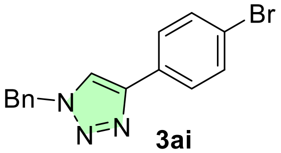

3ai

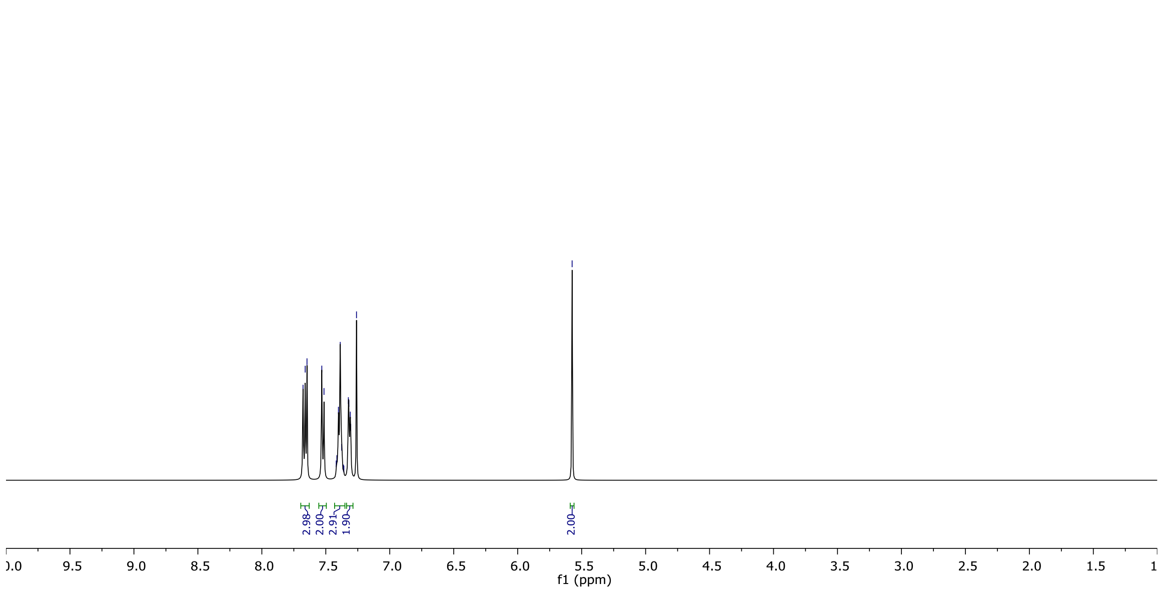

^{1}H NMR of 1-Benzyl-4-(4-bromophenyl)-1H-1,2,3-triazole (3ai)

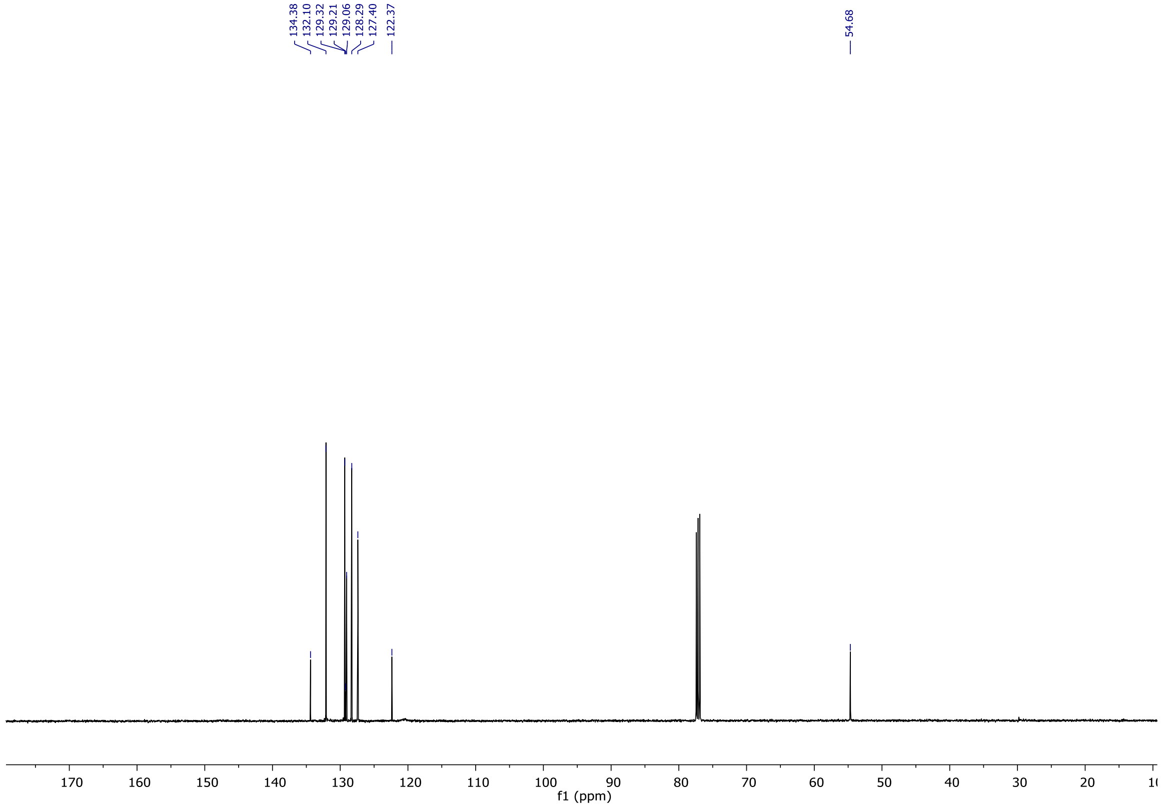

^{13}C NMR of 1-Benzyl-4-(4-bromophenyl)-1H-1,2,3-triazole (3ai)

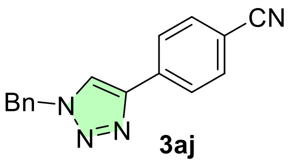

3aj

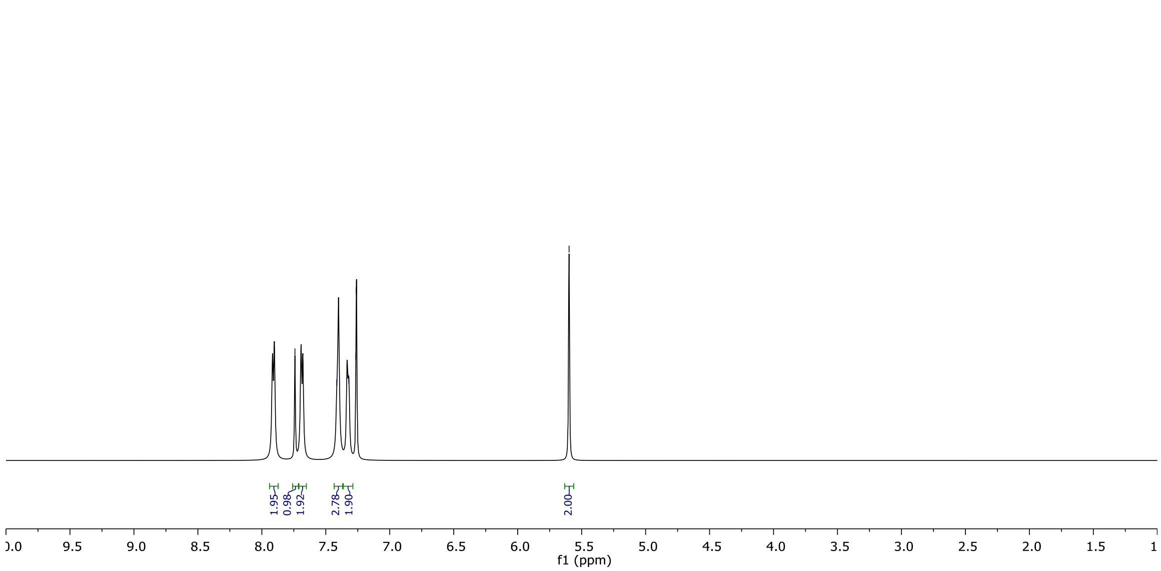

^{1}H NMR of 4-(1-Benzyl-1H-1,2,3-triazol-4-yl)benzonitrile (3aj)

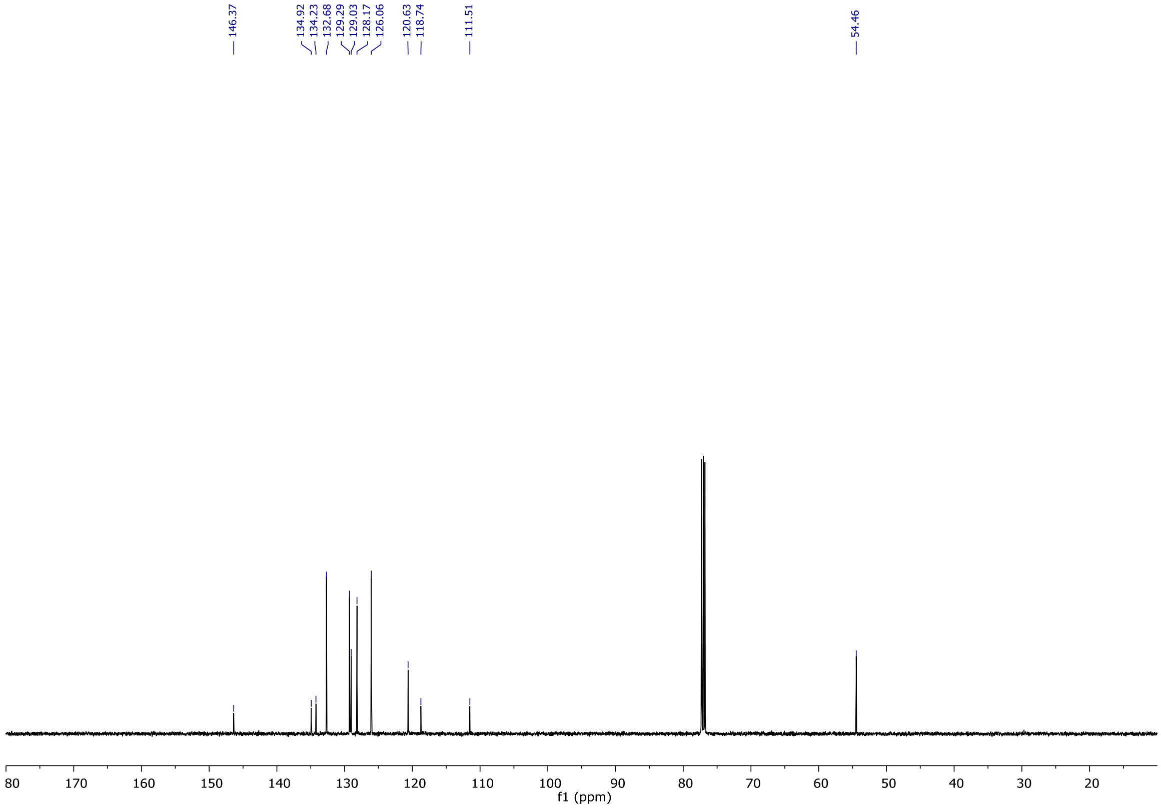

^{13}C NMR of 4-(1-Benzyl-1H-1,2,3-triazol-4-yl)benzonitrile (3aj)

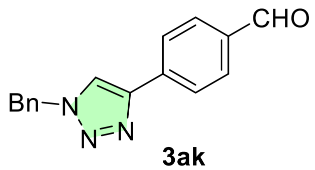

3ak

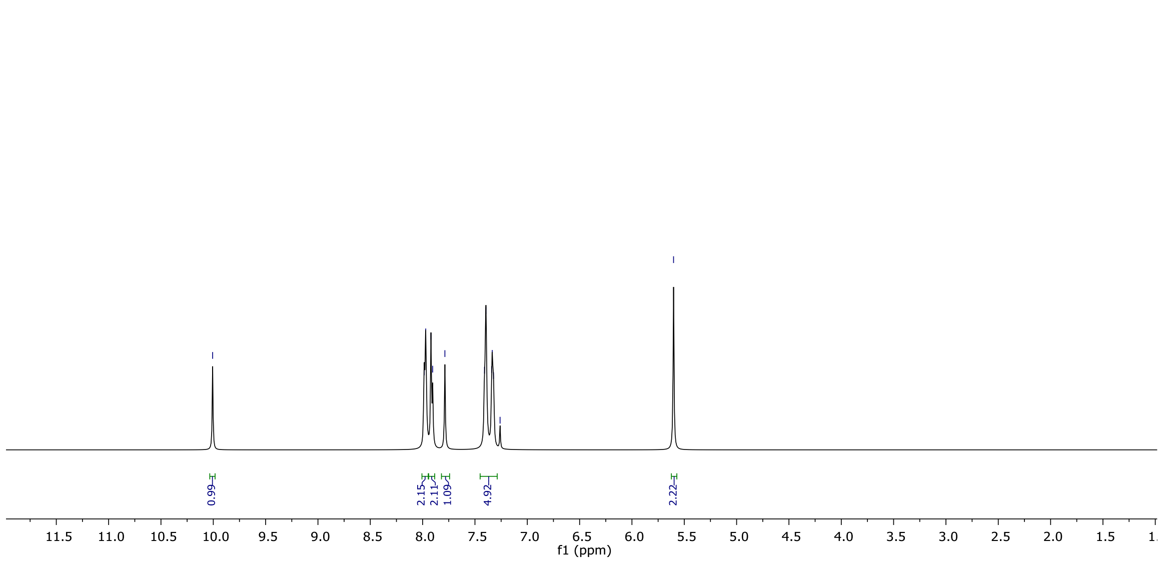

^{1}H NMR of 4-(1-Benzyl-1H-1,2,3-triazol-4-yl)benzaldehyde (3ak)

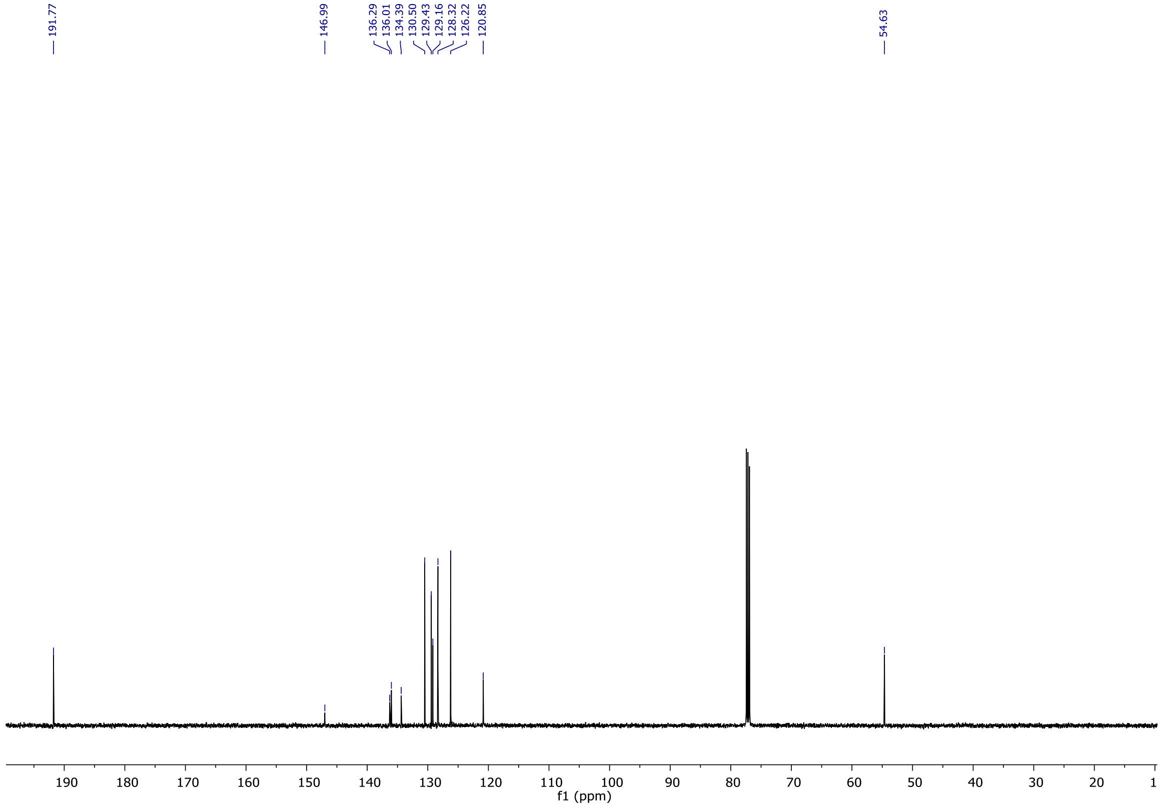

^{13}C NMR of 4-(1-Benzyl-1H-1,2,3-triazol-4-yl)benzaldehyde (3ak)

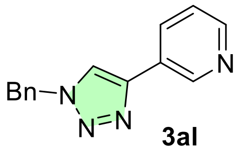

3al

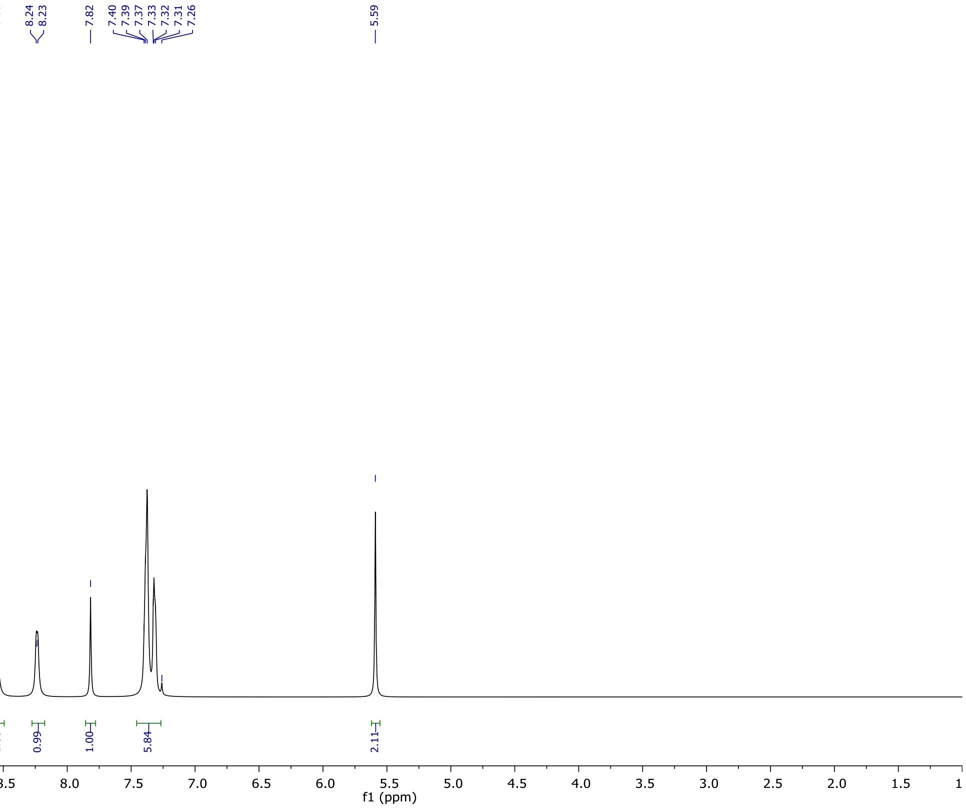

^{1}H NMR of 3-(1-Benzyl-1H-1,2,3-triazol-4-yl)pyridine (3al)

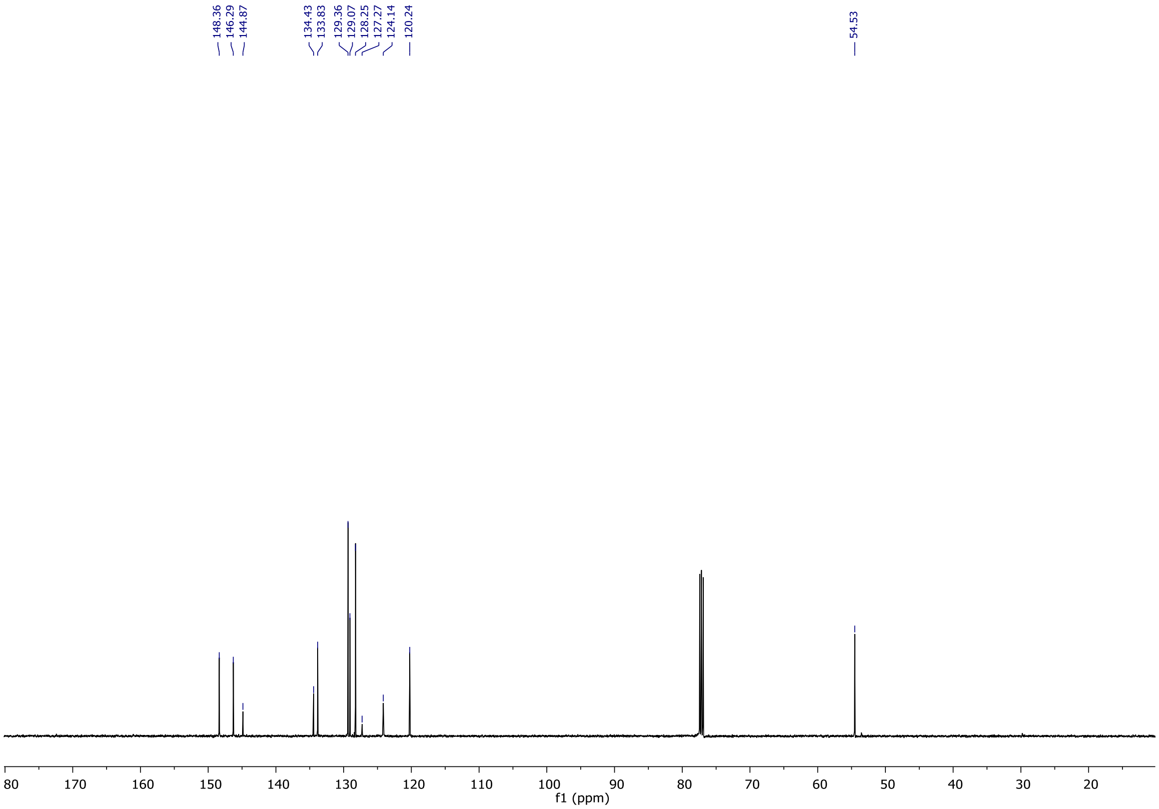

^{13}C NMR of 3-(1-Benzyl-1H-1,2,3-triazol-4-yl)pyridine (3al)

3am

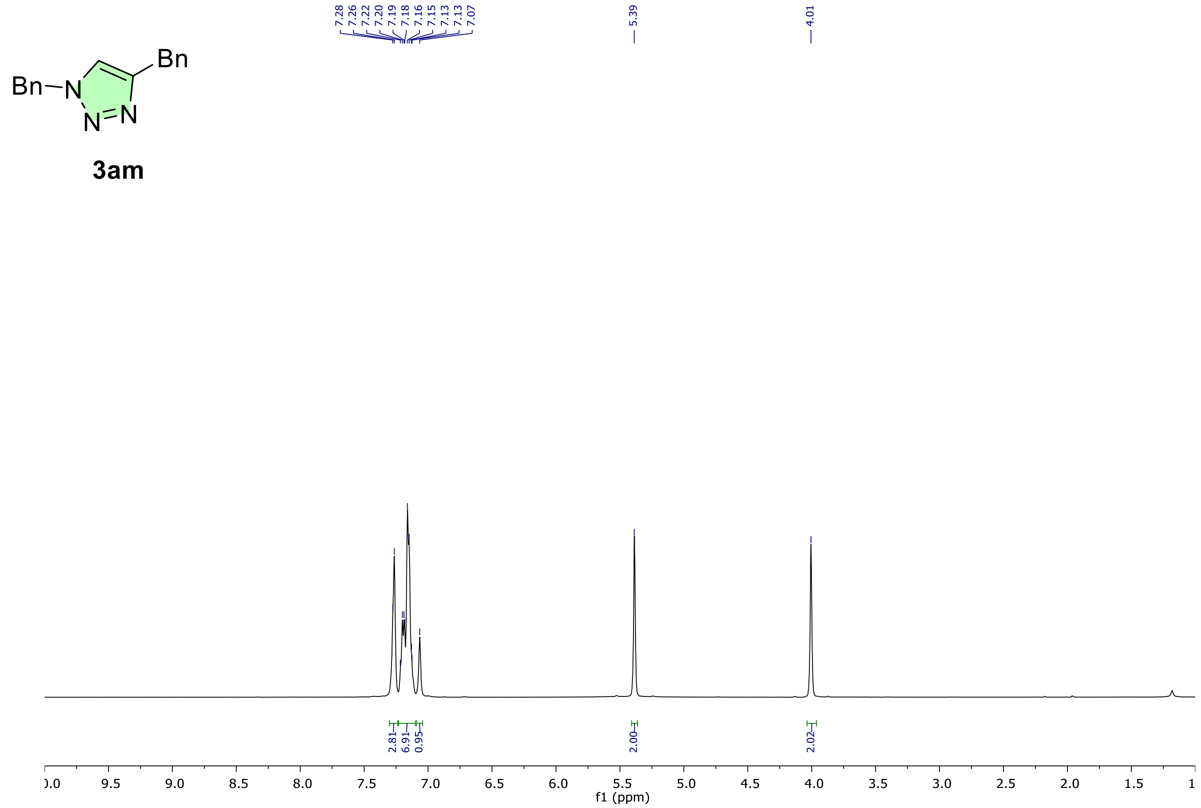

^{1}H NMR of 1,4-Dibenzyl-1H-1,2,3-triazole (3am)

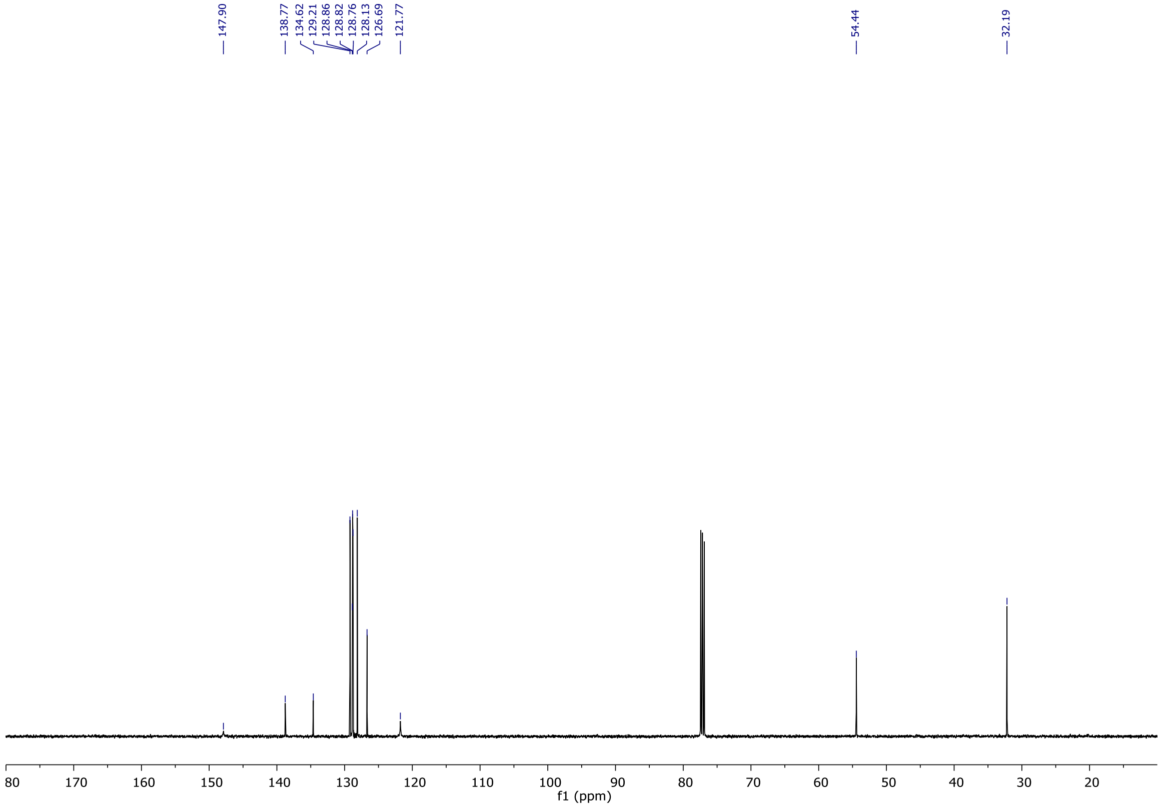

^{13}C NMR of 1,4-Dibenzyl-1H-1,2,3-triazole (3am)

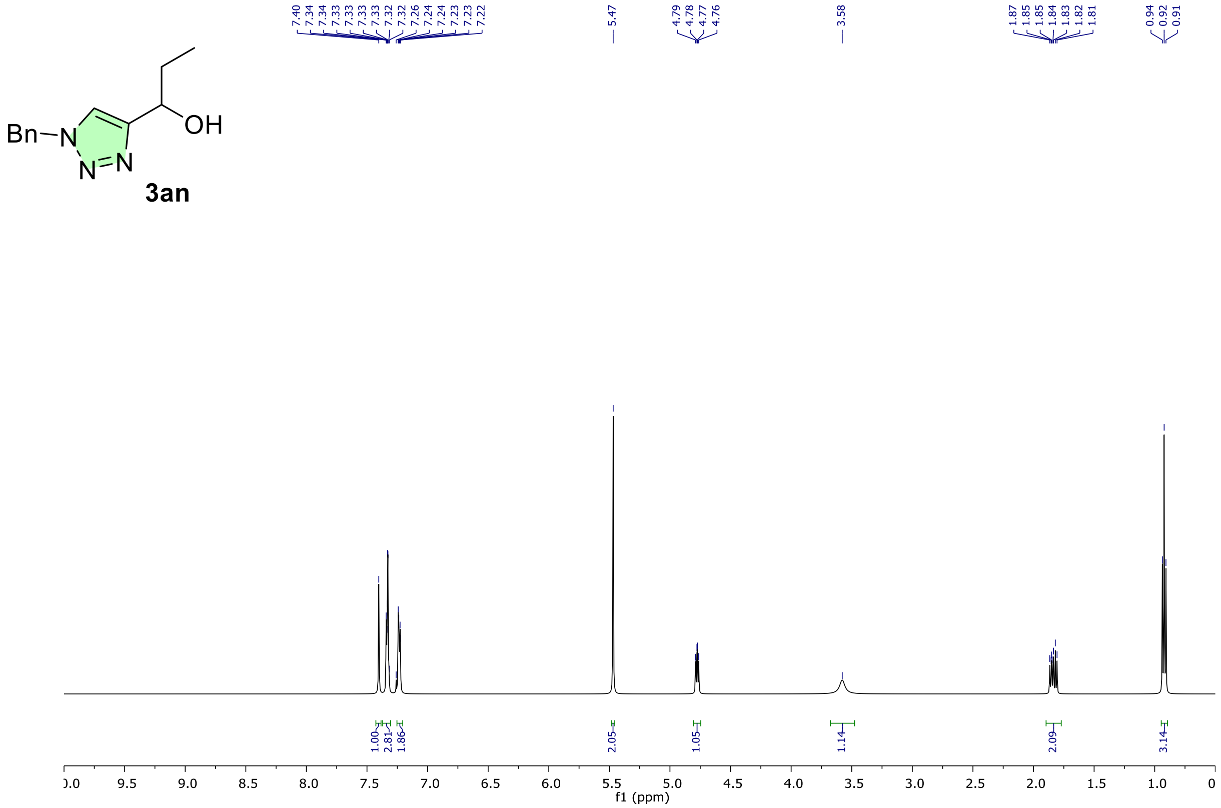

^{1}H NMR of 1-(1-Benzyl-1H-1,2,3-triazol-4-yl)propan-1-ol (3an)

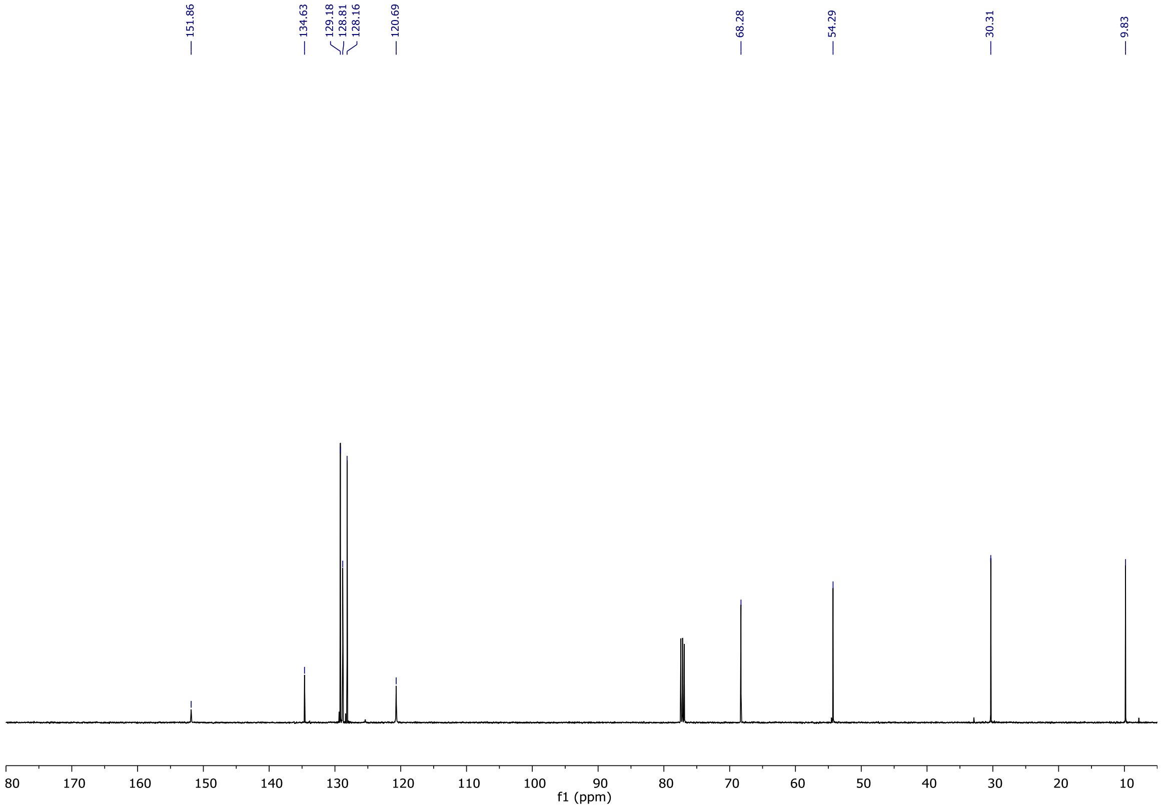

^{13}C NMR of 1-(1-Benzyl-1H-1,2,3-triazol-4-yl)propan-1-ol (3an)

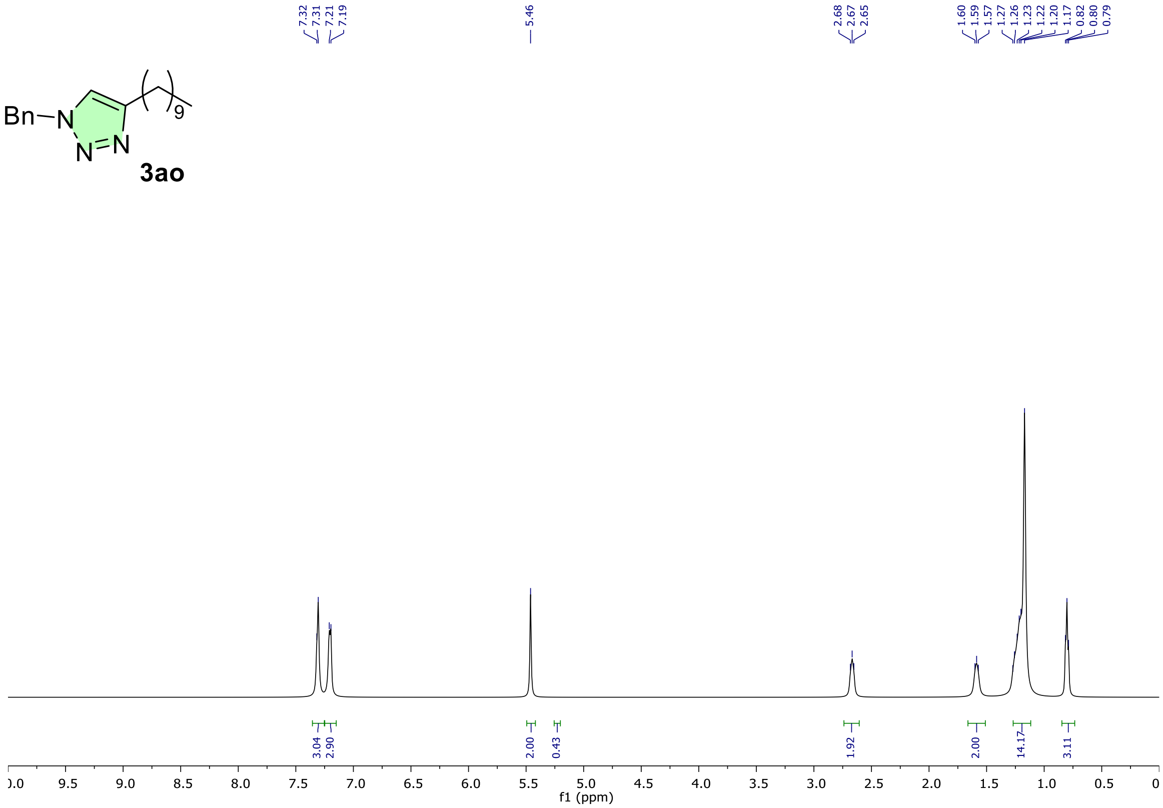

^{1}H NMR of 1-Benzyl-4-decyl-1H-1,2,3-triazole (3ao)

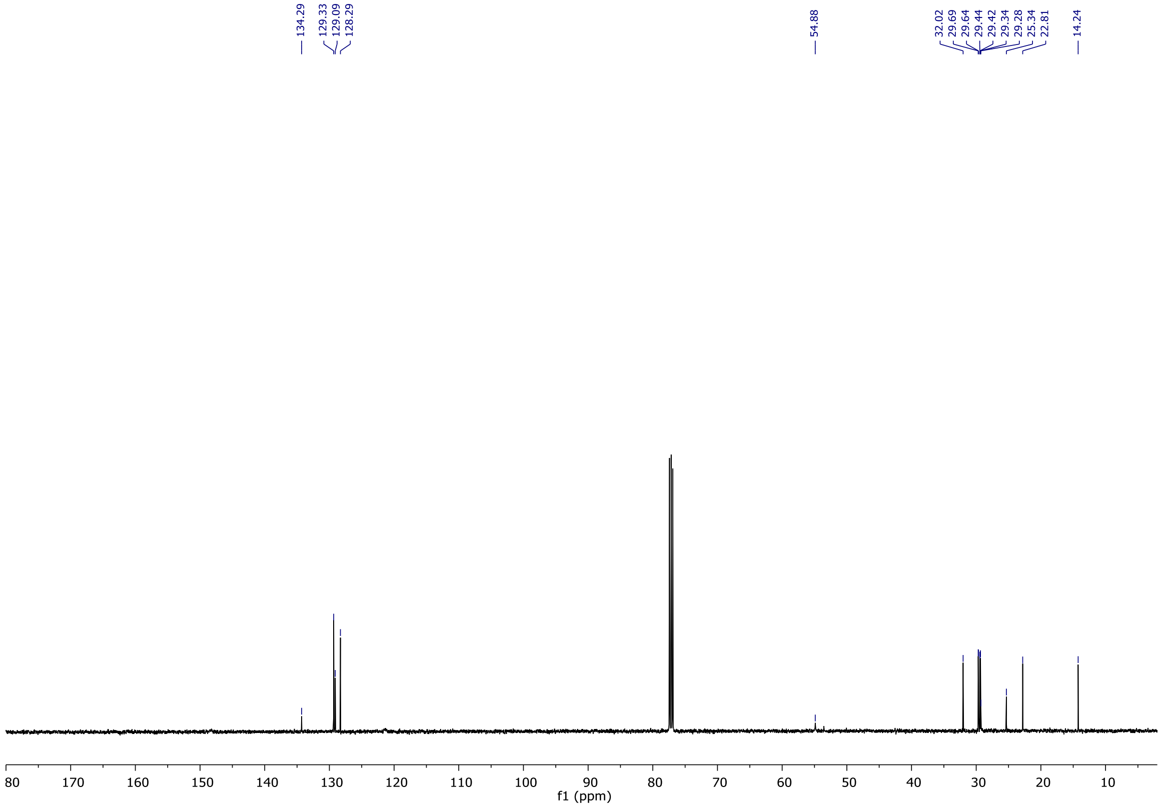

^{13}C NMR of 1-Benzyl-4-decyl-1H-1,2,3-triazole (3ao)

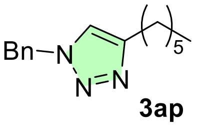

3ap

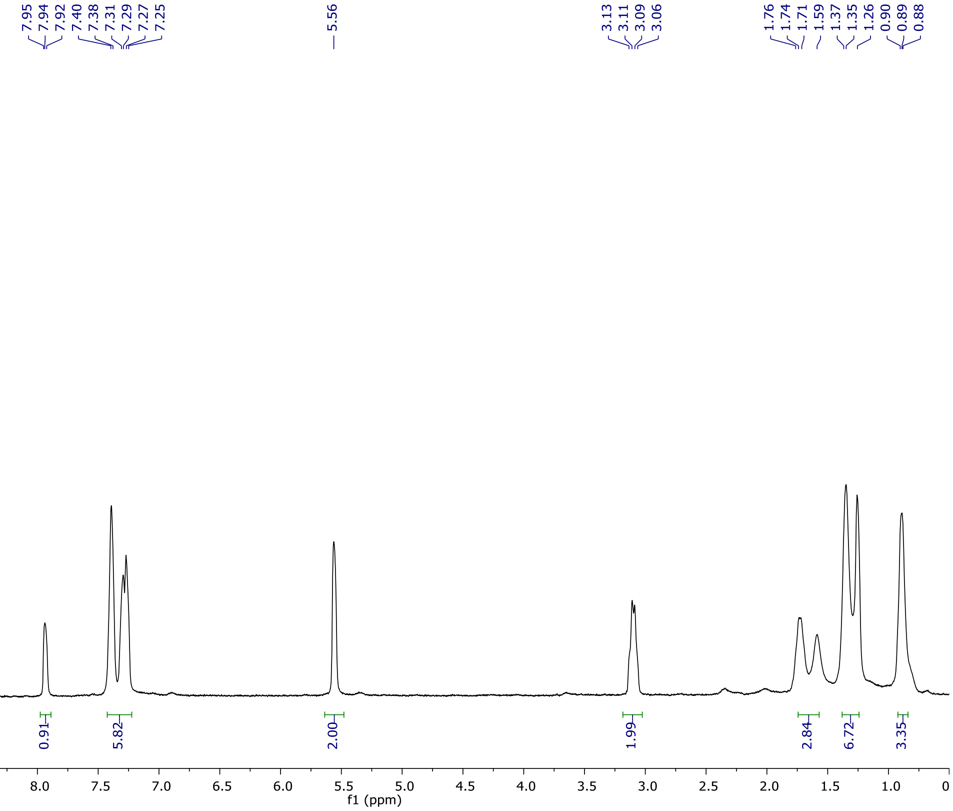

^{1}H NMR of 1-Benzyl-4-hexyl-1H-1,2,3-triazole (3ap)

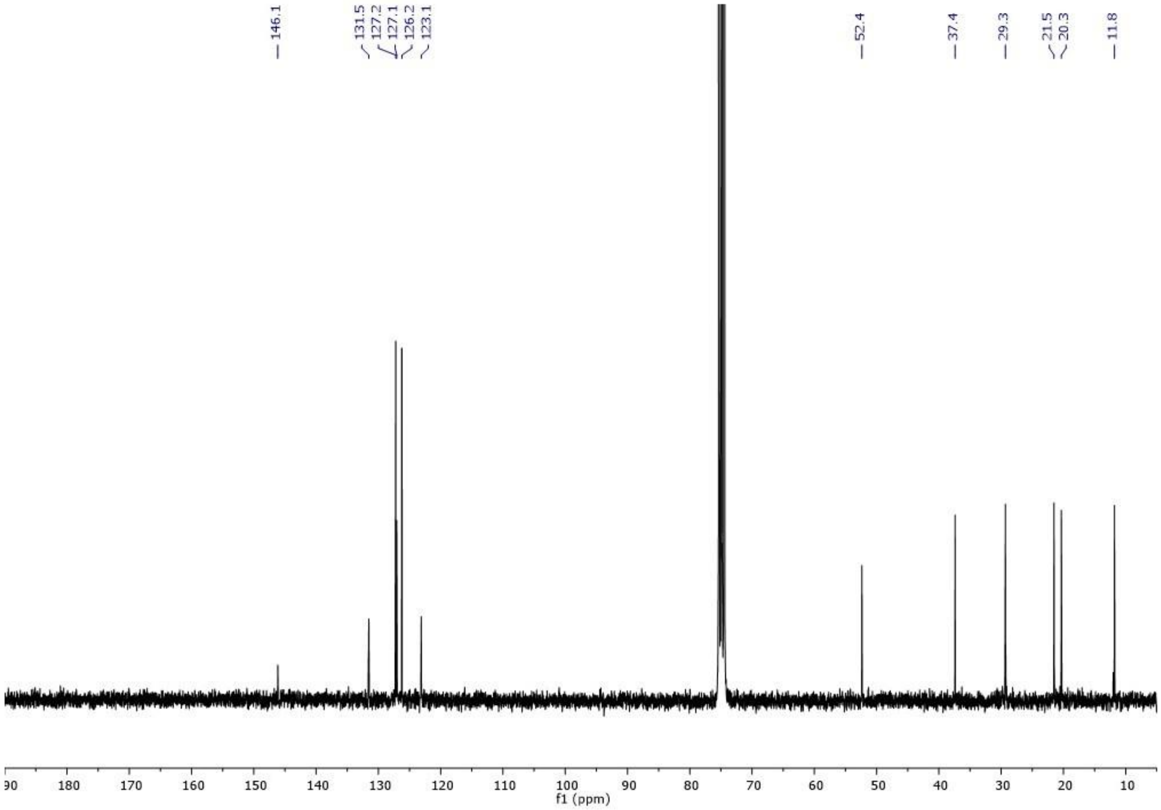

^{13}C NMR of 1-Benzyl-4-hexyl-1H-1,2,3-triazole (3ap)

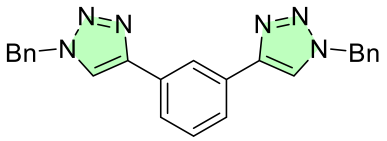

3aq

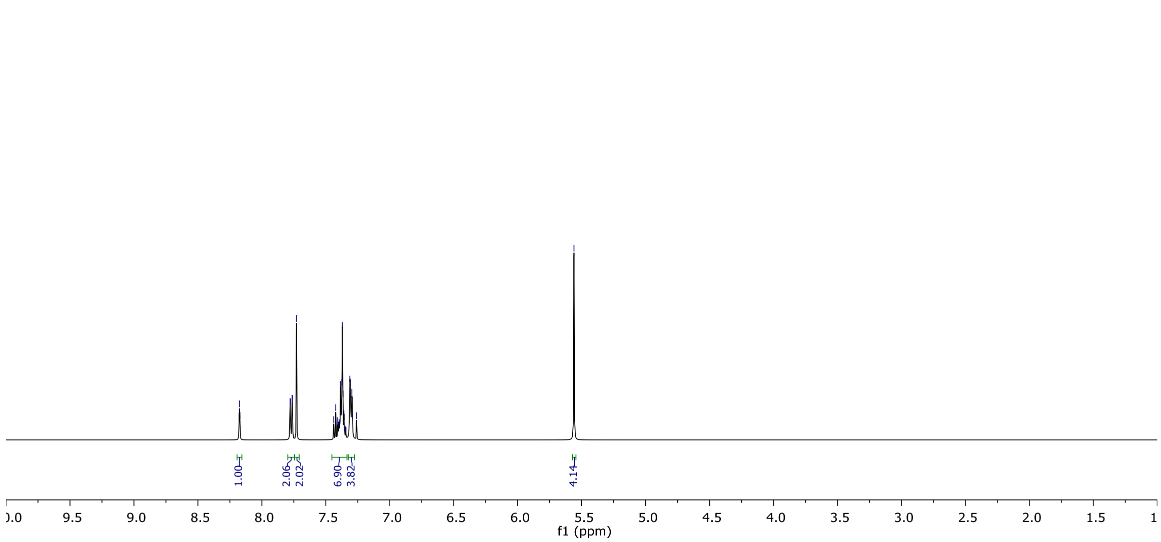

^{1}H NMR of 1,3-Bis(1-benzyl-1H-1,2,3-triazol-4-yl)benzene (3aq)

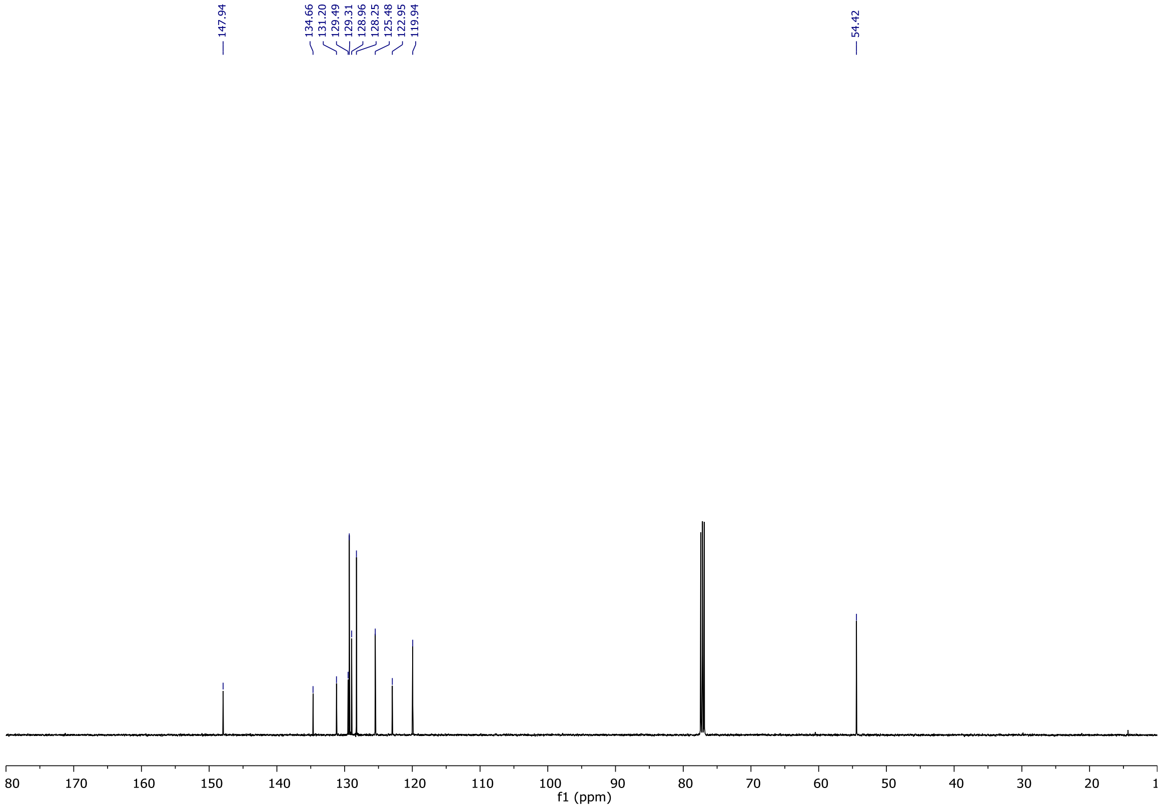

^{13}C NMR of 1,3-Bis(1-benzyl-1H-1,2,3-triazol-4-yl)benzene (3aq)

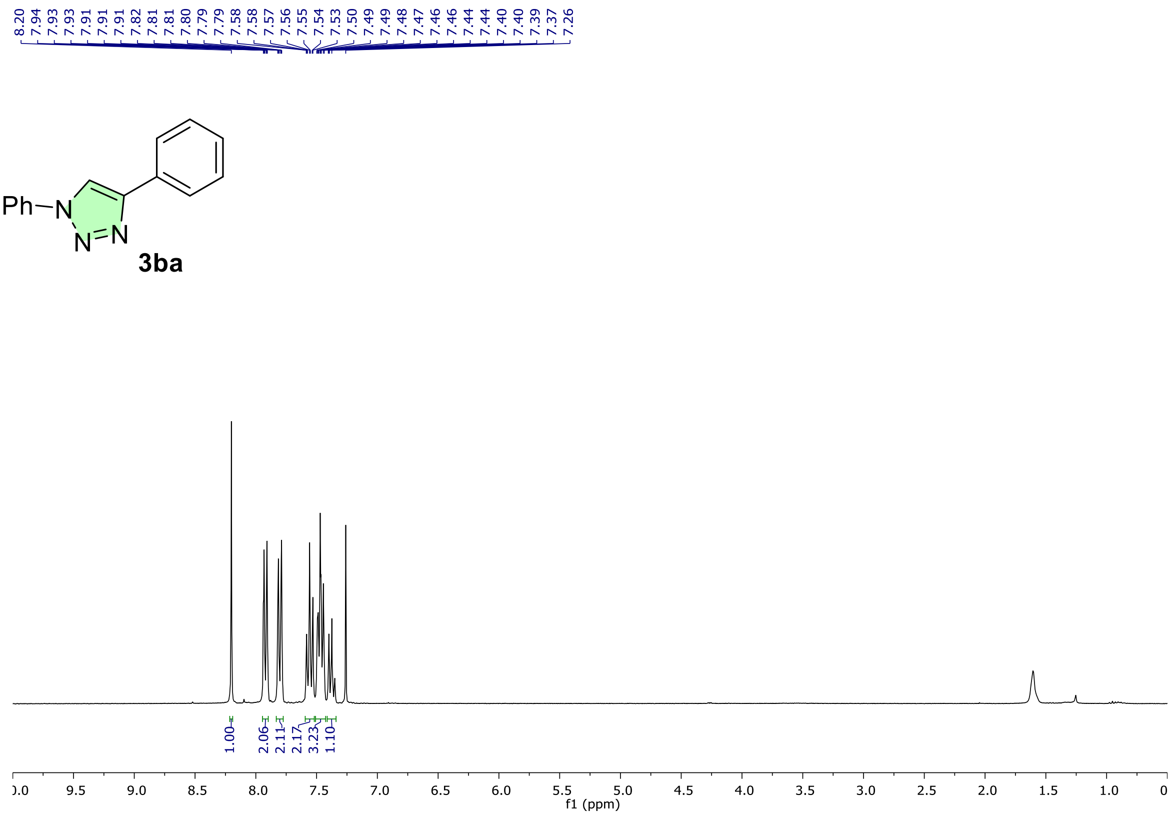

^{1}H NMR of 1-Phenyl-4-phenyl-1H-1,2,3-triazole (3ba)

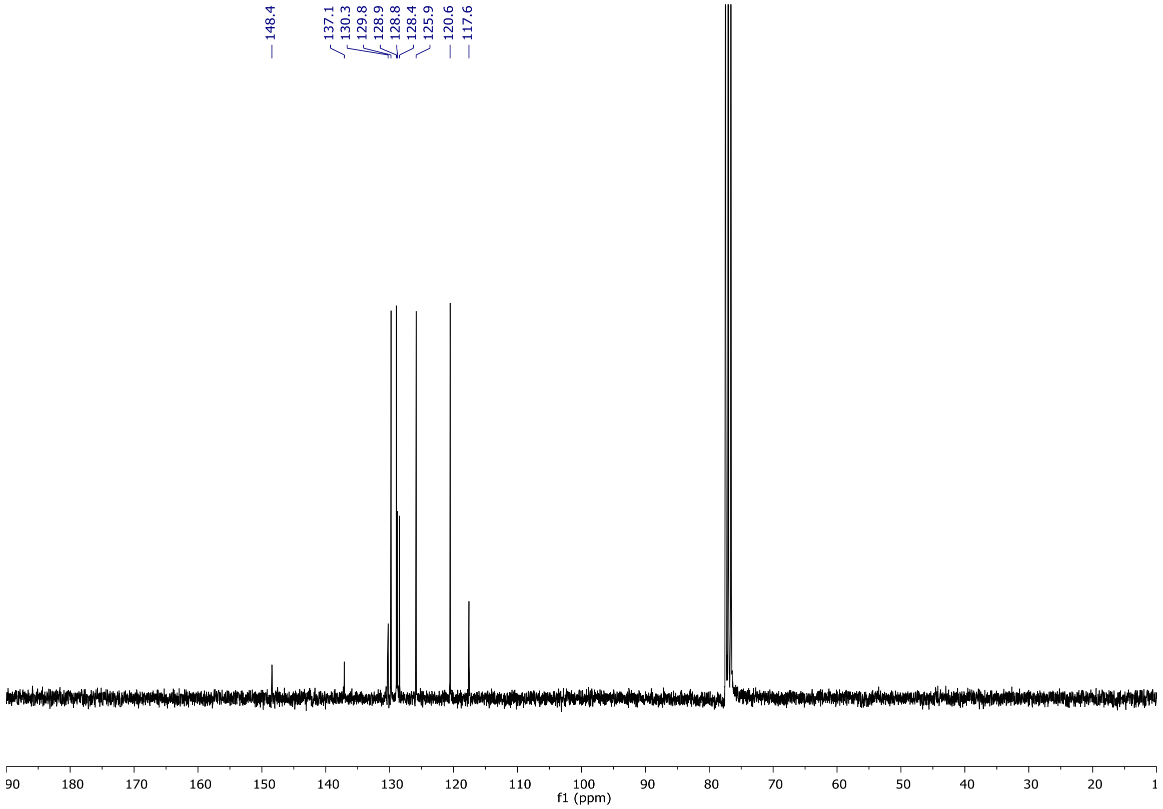

^{13}C NMR of 1-Phenyl-4-phenyl-1H-1,2,3-triazole (3ba)

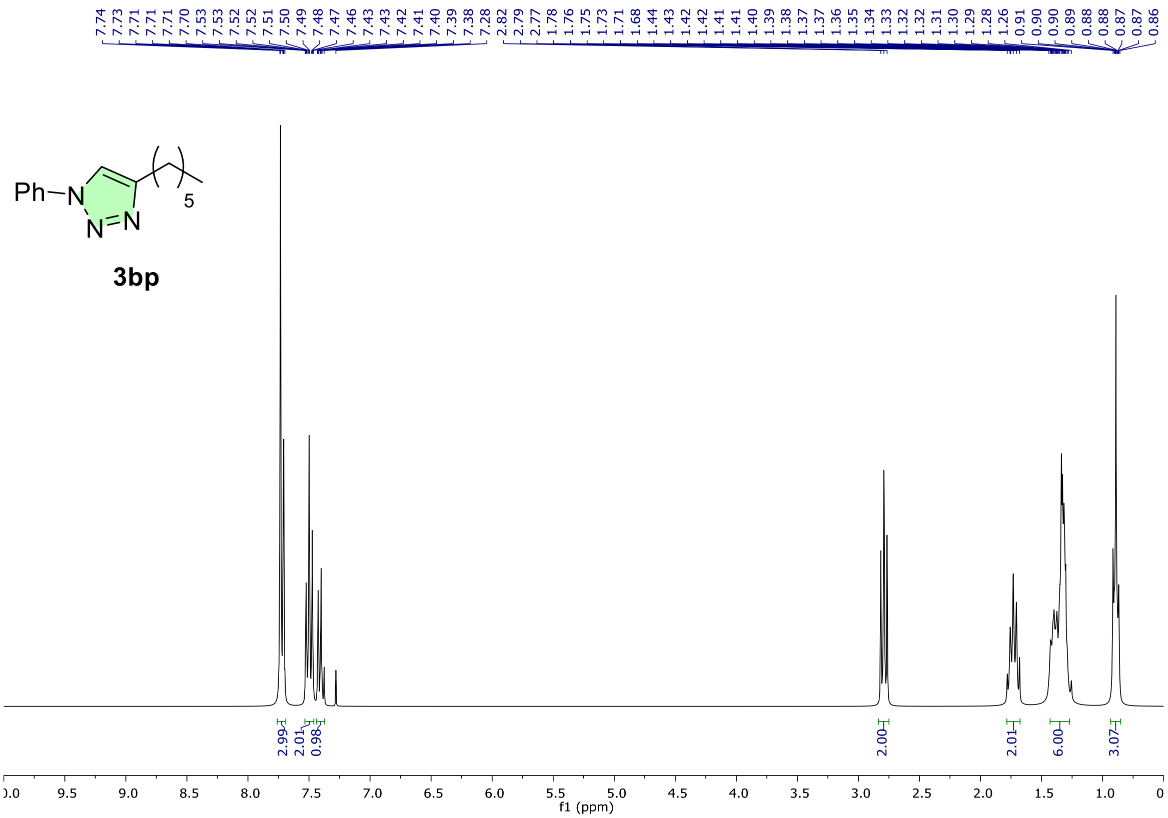

^{1}H NMR of 1-Phenyl-4-hexyl-1H-1,2,3-triazole (3bp)

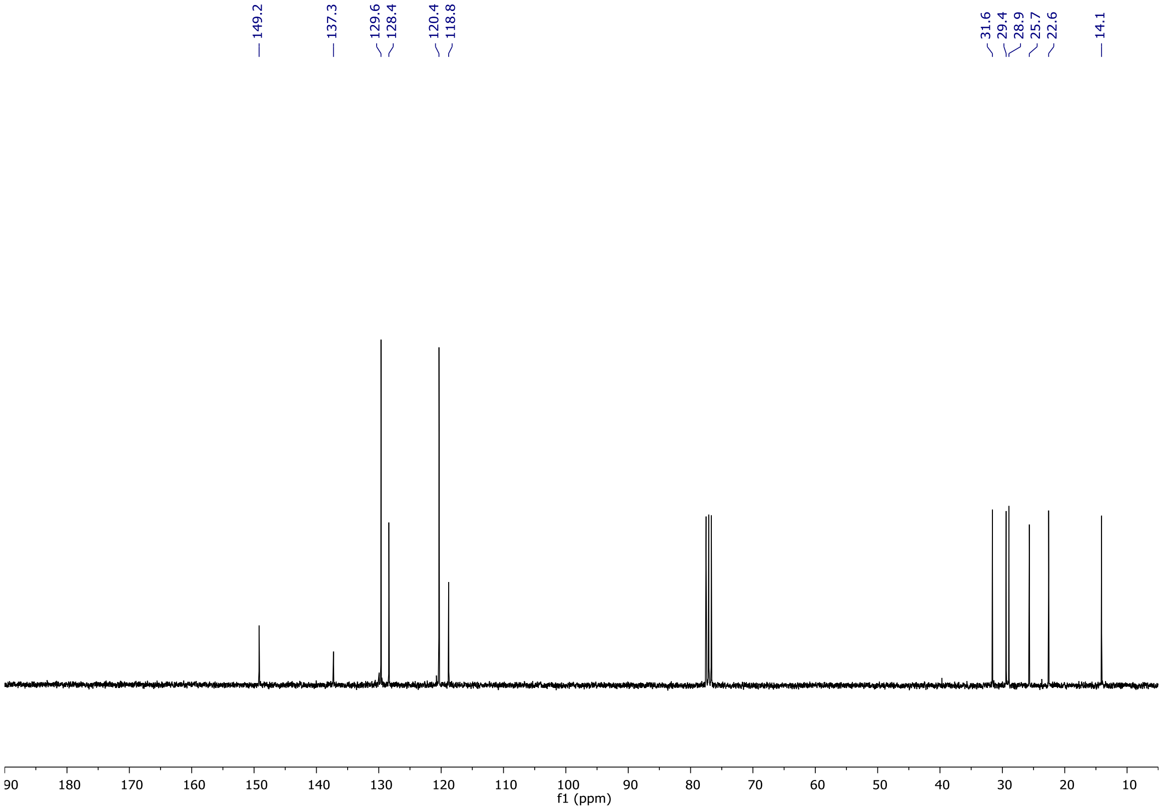

^{13}C NMR of 1-Phenyl-4-hexyl-1H-1,2,3-triazole (3bp)

8 References

1. L. Bahsis, E.-H. Ablouh, H. Anane, M. Taourirte, M. Julvee, S.-E. Stiriba. *RSC Adv.*, **2020**, 10, 32821.
2. K. Rajender Reddy, K. Rajgopal, M. Lakshmi Kantam. *Catal. Lett.*, **2007**, 114, 36.
3. L. Bahsis, H. B. El Ayouchia, H. Anane, K. Benhamou, H. Kaddami, M. Julve, S.-E. Stiriba. *Int. J. Biol. Macromol.*, **2018**, 119, 849.
4. B. H. Mandal, M. L. Rahman, M. M. Yusoff, K. F. Chong, S, M. Sarkar. *Carbohydr. Polym.*, **2017**, 156, 175.
5. R. B. N. Baig, R.S. Varma, R. S. *Green Chem.*, **2013**, 15, 1839.
6. E.-H. Ablouha,L. Bahsis, H. Sehaqui, H. Anane, M. Julved, S.-E. Stiribad, M. El Achabya. *Sustain. Chem. Pharm.*, **2022**, 30, 100837.
7. F. Alonso, Y. Moglie, G. Radivoy, M. Yus. *Eur. J. Org. Chem.*, **2010**, 1875.
8. X. Li, S. Ajmal, P. Fang, X. Zhou, M. Lu, S. Li, P. Chen, M. Zhu. *New J. Chem.*, **2024**, 48, 13859.
9. B. N. Diehl, J. Hamdi, J. Do, L. Cruz, M. Spengeman, F. R. Fronczek, M. L. Trudell. *ChemNanoMat*, **2024**, 10, e202400212.
10. G. Vilé, G. Di Liberto, S. Tosoni, A. Sivo, V. Ruta, M. Nachtegaal, A. H. Clark, S. Agnoli, Y. Zou, A. Savateev, M. Antonietti, G. Pacchioni. *ACS Catal*. **2022**, 12, 5, 2947.
11. E. Ozkal, P. Llanes, F. Bravo, A. Ferrali, M. A. Pericàs. *Adv. Synth. Catal.*, **2014**, 356, 857.
12. P. Ren, Q. Li, T. Song, Z. Wang, K. Motokura, Y. Yang. *ChemCatChem*, **2021**, 13, 3960.
13. M. Kr. Barman, A. K. Sinha, S. Nembenna. *Green Chem.*, **2016**, 18, 2534
14. N. Mukherjee, S. Ahammed, S. Bhadra, B. C. Ranu. *Green Chem.*, **2013**, 15, 389.
15. Q. Jia, G. Yang, L. Chen, Z. Du, J. Wei, Y. Zhong, J. Wang. *Eur. J. Org. Chem.*, **2015**, 3435.
16. F. Alonso, Y. Moglie, G. Radivoy, M. Yus. *Org. Biomol. Chem.*, **2011**, 9, 6385.
17. P. Gogoi, T. Saikia, R. Hazarika, A. Garg, K. Deori, D. Sarma. ACS Sustainable Chem. Eng. **2023**, 11, 42, 15207.
